# Genomic and Multi-Omics Analysis of *Phlebopus portentosus*: Effects of Cultivation on Secondary Metabolites

**DOI:** 10.3390/jof11040323

**Published:** 2025-04-18

**Authors:** Zujiang Kang, Xiaolong Yuan, Chuanguang Zhang, Yi Wang, Lu Li, Yuan Zheng

**Affiliations:** 1 College of Forestry, Southwest Forestry University, Kunming 650224, China; 2 Yunnan Key Laboratory of Biodiversity of Gaoligong Mountain, Yunnan Academy of Forestry & Grassland Science, Kunming 650204, China; yuanxiaolong@yafg.ac.cn (X.Y.); zhangchuanguang@yafg.ac.cn (C.Z.); 3 College of Biological and Food Engineering, Southwest Forestry University, Kunming 650224, China

**Keywords:** *Phlebopus portentosus*, secondary metabolite, metabolome analysis, transcriptome sequencing, transcriptional regulation, polyketide synthase, terpene synthase, multi-omics association

## Abstract

*Phlebopus portentosus* is an edible and medicinal ectomycorrhizal mushroom with delicious and high nutritional value. However, the mechanism of secondary metabolite biosynthesis in *P. portentosus* is still unclear. In this study, the genomics, metabolomics, and transcriptomics were integrated to reveal the biosynthesis mechanism of secondary metabolites in *P. portentosus* under different cultivation conditions. The 31.4 Mb genome of *P. portentosus* YAF023 with 15 scaffolds was assembled by Illumina and Nanopore sequencing and annotated, and 206 cytochrome P450s, 201 carbohydrate-active enzymes, 186 transcription factors, 18 terpene synthases (TPSs), and 5 polyketide synthases (PKSs) were identified. Multi-omics analysis showed that *PpPKS1* is probably involved in the biosynthesis of Ethyl orsellinate; *PpPKS2* and *PpPKS5* are probably involved in the synthesis of 6-Methylsalicylic acid and Cytochalasin Z5, respectively; *PpTRI5* was involved in the tetracyclic sesquiterpene *β*-type trichodiene compounds; and *PpSTCs* was involved in the synthesis of β-copaene analogs or derivatives. Co-expression network analysis and binding site prediction of the promoter regions suggested that *PpHOX4* and *PpHSF1* regulated the gene expression of *PpPKS1*, and *Ppzf-C2H2 32* and *PpHSF5* regulated the gene expression of *PpSTCs 8*, and *PpSTCs 3,* respectively. This study will provide an important foundation for further development and utilization of secondary metabolites of *P. portentosus*.

## 1. Introduction

Edible mushrooms have long been used as food due to their richness in protein, dietary fiber, vitamins, and minerals [1]. Essential amino acids that humans cannot synthesize are found in mushrooms, such as *Agaricus bisporus*, and *Boletus edulis*, and in other wild edible mushrooms [2]. *Phlebopus portentosus* (Berk. and Broome) Boedijin is an edible ectomycorrhizal (ECM) mushroom, with a delicious taste and high nutritional value [3,4]. In the Xishuangbanna region of Yunnan, China, *P. portentosus* is a local favorite wild mushroom with a price of 60–100 RMB/kg (USD 9–14) [5,6]. *P. portentosus* is widely distributed in the tropical regions of China and Thailand, especially in Yunnan, Guangxi, and Hainan [7]. It is locally known as “black Boletus” because of its dark-colored fruiting bodies. *P. portentosus* is found growing on both forest trees and fruit trees. Because it can grow either with various trees or without a host tree, it is reported that it may not be an obligate ectomycorrhizal fungus [8]. Recent years have witnessed a significant decline in the production of the wild *P. portentosus*, primarily attributed to unsustainable commercial harvesting. Therefore, a different cultivation of *P. portentosus* has been carried out [7]. For example, cultivation of *P.portentosus* associated with *Sesbania javanica* has been developed [8]. Technologies for the semi-artificial cultivation and artificial cultivation of *P. portentosus* in mushroom houses have also been developed; it is the first edible boletus species that can be cultivated artificially [9,10]. *P. portentosus* is rich in nutrients, including polysaccharides, proteins, dietary fiber, and mineral elements [11]. Recent research shows that *P. portentosus* can also produce many bioactive compounds such as polysaccharides with hypoglycemic activity [12], pyrrole alkaloids with neuroprotective effects [13], 9′-hydroxyphenyl pulvinone and β-carboline alkaloid with antimicrobial and anticancer bioactive [14,15], and polyphenols with antioxidant activity [16].

So far, approximately 15,000 types of secondary metabolite (SM) natural products have been discovered, which can be classified into polyketides, non-ribosomal peptides, terpenoids, and sterols according to their structural components, and most of them belong to polyketides. Currently, a large number of studies indicate that the main functions of polyketides include antioxidant, antitumor, immunosuppressive, antibacterial, and antiparasitic properties. [17,18]. Currently, there is no research on *P. portentosus* polyketides, and research on Boletinellaceae polyketides is also relatively scarce. Polyketides include many medicinally active compounds, such as Lovastatin, which has the effect of lowering blood cholesterol [19], orsellinic acid and its derivatives, which have biological anti-inflammatory, antimicrobial, antitumor, antioxidant, antiviral, and immunosuppressive activity [20], cytochalasans, an SM with antibacterial, antiviral, and bacteriostatic activities produced by *Aspergillus flavus* [21], highly toxic SMs of aflatoxin produced by *A. flavus*, *A. parasiticus*, and *A. nomius* [22].

The biosynthesis of polyketides is mainly catalyzed by polyketide synthase (PKS) and post-modifying enzymes. PKS is the key enzyme in the synthesis of polyketides, which catalyzes the sequential decarbonylation and recurrent condensation of multiple lipoyl coenzyme A to produce polyketides. PKS can be divided into three types according to its protein structure and catalytic mechanism, including Type I, Type II, and Type III. Type I PKS includes Type I modular PKS and Type I iterative PKS. A typical Type I modular PKS is a multifunctional complex enzyme consisting of modules with catalytic domains containing a *β*-ketoacyl thioester synthase (KS) domain, an acyltransferase (AT) domain, and an acyl carrier protein (ACP) domain, which form the smallest catalytic module PKS. In addition, there are other structural domains in Type I PKS, such as ketoreductase (KR), which are capable of modifying the extended carbon atoms of the main chain, dehydratase (DH), enoylreductase (ER), c-methyltransferase (CMeT), and thioesterase (TE) [23]. Fungal Type I PKSs can be further classified into three subgroups based on the degree of reduction of the *β*-keto group: highly reducing PKSs (HR-PKSs), in addition to the basic structural domains, an intact reduction loop KR, DH, and ER, as well as a methyl transferase (MeT), partially reducing PKSs (PR-PKSs) that do not have a complete reduction loop, nonreducing PKSs (NR-PKSs) that lack KR, DH, and ER domains, but in addition to KS, AT, ACP, and TE domains, usually have starter acyltransferase (SAT) and product template (PT) domains [24].

Terpenoids are a large group of natural products with diverse structures and various biological activities. Macrofungi can produce many triterpenoids and sesquiterpenoids and meroterpenoids with anticancer, antioxidant, antiviral, and antimicrobial bioactivity [25,26,27,28,29]. Several bioactive triterpenoids were obtained from medicinal fungi by fermentation [30]. Different substrates and cultivating conditions will affect the yield of triterpenes [31,32]. Terpene synthases (TPSs) are a class of key enzymes in the biosynthesis of terpenoids, which play important functions in terpenoid biosynthesis. Different TPSs determine the diversity of terpene carbon skeletons and also determine their functional diversity [33]. TPSs can be divided into three classes based on the mode of formation of the initiating carbon positive ion [34]. Class I terpenoid cyclase includes monoterpene, sesquiterpene, and diterpene cyclase, which remove the pyrophosphate group of the substrate by ionization of metal ions (Mg^2+^, Mn^2+^); Class II terpenoid cyclase includes diterpene, triterpene, and sesquiterpene cyclase, which remove the pyrophosphate group of the substrate by protonation of a carbon–carbon double bond formed by aspartate side chains. The catalytic structural domains of Class I terpenoid cyclase are an aspartic acid-rich region (DDXXD/[Mg^2+^]3) and NSE/DTE. which mainly remove the pyrophosphate group of the substrate by ionization; the catalytic structural domains of Class II terpenoid cyclase are also an aspartate-rich region (DXDD), which remove the pyrophosphate group of the substrate mainly through protonation [35].

Several genomes of *P. portentosus* have been reported [36,37,38]. Metabolomic and transcriptomic analysis was also used to reveal the polyphenol biosynthesis in *P. portentosus* [16]. However, there are no reports about the effects of cultivation on secondary metabolites of *P. portentosus* that integrate genomic and multi-omics analysis. In this study, whole-genome sequencing, functional annotation, and SM analysis of *P. portentosus* YAF023 were performed by a next-generation Illumina NovaSeq combined with a third-generation Oxford Nanopore Technologies sequencing platform. On this basis, the SM-related PKS and TPS genes of *P. portentosus* were further uncovered and analyzed by combining the results of LC-MS/MS analysis and RNA-Seq analysis under three different cultivation conditions. And based on the whole-genome data of *P. portentosus*, its related family TFs such as Zinc finger (ZnF), high heat shock factor (HSF), homeodomain (HOX), transcription factor TFIIB repeat (TFIIB), and Fungal_trans domain (FTD) were identified and bioinformatically analyzed. Additionally, the expression of PKS, TPS, and transcription factors (TFs) was analyzed in *P. portentosus* under different cultivation conditions, and the transcription factors regulating the expression of TPS and PKS gene were also explored. This study provides further reference for the mining of SM genes and their related metabolic transcriptional regulation studies in *P. portentosus*.

## 2. Materials and Methods

### 2.1. Source of Strains and Culture Media

The *P. portentosus* YAF023 was isolated from the fruiting body grown in the underwood of *Pinus yunnanensis*, and it was collected from Panzhihua, Sichuan Province [38]. It has been deposited in the Yunnan key laboratory of biodiversity of Gaoligong mountain, Yunnan academy of forestry and grassland sciences in Kunming, and the China center for type culture collection (deposit number: CCTCC M 20232694) in Wuhan, China. *P. portentosus* YAF023 was inoculated in PDA, cultured in a constant-temperature incubator at 26 °C and 60% humidity for 25 days, and then stored in a refrigerator (Harer, Qingdao, China) at 4 °C. During the experiment, 0.5 cm^2^ of mycelium was uniformly scooped from the edge of *P. portentosus* mycelium and inoculated in different media: PDA medium for solids and incubated at 28 °C for 25 days, and BDMY medium for liquid culture at 28 °C for 25 days on a rotary shaker at 150 rpm.

Solid PDA formulation was as follows: boiled potato juice (200 g/L), KH_2_PO_4_ (1 g/L), MgSO_4_ (0.5 g/L), yeast powder (5 g/L), vitamin B1 (0.1 g/L), and agar (16 g/L) [38]. BDMY liquid media formulation was as follows: Difco™ malt extract broth (7.5 g/L), malt/yeast extract (5 g/L), yeast powder (1 g/L). The cultivation process for BDMY liquid with valproic acid: after 25 days of growth in BDMY liquid culture, valproic acid (10 μmol/L) was added and incubated in a shaker for 10 days [28].

The solid PDA medium used in subsequent articles is abbreviated as L016_PDA, the liquid BDMY medium is abbreviated as L016, and the liquid-added valproic acid medium is abbreviated as L016_BWS.

### 2.2. Genome Sequencing 

After 25 days of liquid culture, the *P. portentosus* mycelium was extracted and subjected to high-throughput sequencing at Shanghai Personalbio Biotechnology Co., Ltd., Shanghai, China. Using the Whole-Genome Shotgun (WGS) strategy, the *P. portentosus* gene library was constructed with 400 bp insert fragments. The Illumina NovaSeq sequencing platform (Illumina, CA, USA) and Nanopore PromethION sequencing platform (Oxford Nanopore Technologies, Oxford, UK) were used for short- and long-read sequencing, respectively. The low-quality data were filtered from the sequenced sequences to obtain pass reads, and based on the filtered data, assembly and subsequent analysis were performed to ultimately obtain the complete genome sequence. The raw data were saved in FASTQ format, and the software fast (version 0.21.0) was used to filter the raw reads, discarding low-quality reads. The software Oxford Nanopore GUPPY (version 0.3.0) was used to perform quality assessment on the raw data, filtering out fail reads with sequencing quality values Q < 7, resulting in pass reads. Using the reads obtained from sequencing, K-mer-based analysis methods were used to estimate genome size and heterozygosity, among other factors [39]. Preliminary genome assembly was performed using the software NECAT (https://github.com/xiaochuanle/NECAT; accessed on 6 February 2025) to construct contigs and scaffolds, while preliminary error correction was performed by the software Racon (version: 1.4.11) and Pilon (version: 1.23); the software BUSCO (version: 4.1.2) was used to assess the integrity of genome assembly.

### 2.3. Gene Prediction and Annotation

Using the MAKER (version: 2.31.10) software, the gene sets predicted by various methods were integrated. Firstly, the software RepeatMasker (version: open-4.0.9) was used to annotate repeats based on the RepBase library (https://www.girinst.org/repbase; accessed on 6 February 2025); then, the software RepeatModeler (version: open-1.0.11) was used to build a library based on the de novo prediction of its sequence features; finally, all the repeat prediction results were combined and predicted. Then, we used RepeatModeler (version: open-1.0.11) to build a library based on the de novo prediction of our sequence features, and we also used RepeatMasker (version: open-4.0.9) to compare and predict the repeat sequences; finally, all the results of the repeat prediction were merged and made unredundant to obtain the final genome repeat sequence set. The tRNAscan-SE (version: 1.23) was used for tRNA prediction, the rRNA database for rRNA prediction, and INFERNAL (version: 1.1.2) based on the Rfam database to find ncRNA sequences in the genome. BLAST searches of non-redundant (NR) protein sequences from the NCBI, Kyoto Encyclopedia of Genes and Genomes (KEGG), Gene Ontology (GO), and Clusters of Orthologous Groups (COG/KOG) were performed to annotate the gene products.

### 2.4. Additional Annotation

Using HMMER (version: 3.2.1, filtering parameters E-value < 1 × 10^−18^; coverage > 0.35) software, the protein sequences of non-redundant genes were compared with the carbohydrate-active enzymes (CAZymes) database to predict the presence of CAZymes. Using Diamond blastp (version: 2.9.0; parameters: –evalue 1 × 10^−5^), the predicted protein sequences were compared with the Virulence Factor Database (VFDB), Pathogen–Host Interactions Database (PHI), and Fungal P450 database to predict virulence-related genes, classify pathogen–host interaction phenotypes, and predict cytochrome P450-related genes.

### 2.5. Secondary Metabolite Biosynthesis Gene Cluster Analysis

The antiSMASH online tool (https://antismash.secondarymetabolites.org/; accessed on 6 February 2025) was used to predict gene clusters in the scaffolds of *P. portentosus*. Gene structure prediction was conducted using the FGENESH online tool (www.softberry.com/; accessed on 6 February 2025). The PKS/NRPS analysis tool (nrps.igs.umaryland.edu/; accessed on 6 February 2025) was used to predict gene clusters in contigs containing PKS genes to identify domains. Additionally, Protein BLAST (https://blast.ncbi.nlm.nih.gov/; accessed on 6 February 2025) was used for protein alignment on these contigs.

### 2.6. Cluster Analysis

Protein sequences were aligned in MEGA11 software using the Clustal W program, and phylogenetic analyses were performed using the Maximum Likelihood (ML) method. The number of bootstrap tests was set to 10 and other parameters were kept at default settings.

### 2.7. Prediction of TPS Proteins

Using InterProScan (version 5.44-79.0) [40], the TPS (Terpene Synthase) proteins in *P. portentosus* were identified through their conserved domain associations with the following terms: trichodiene synthase (TRI5), sesquiterpene cyclase (STC), polyprenyl synthetase (PPS), prenyltransferase (Ptase), squalene synthase (SQS), and squalene cyclase (SQCY). The obtained candidate gene sequences were compared with the NCBI protein database for confirmation. Then, multiple sequence comparison was performed using DNAMAN (version 2) software to identify the conserved structural domains. The exon and intron composition of the TPS gene was analyzed and visualized using TBtools software.

### 2.8. Transcription Factor Identification Analysis

Domain files for Myeloblastosis (MYB, PF00249), bZIP transcription factor (bZIP1, PF00170), Basic region leucine zipper (bZIP2, PF07716), basic Helix–Loop–Helix (bHLH, PF00010), Zinc finger, C2H2 type (zf-C2H2, PF00096), Zinc finger, C3HC4 type (RING finger) (zf-C3HC4, PF00097), Zinc finger C-x8-C-x5-C-x3-H type (and similar)(zf-CCCH, PF00642), Fungal Zn(2)-Cys(6) binuclear cluster domain(Zn_clus, PF00172), transcription factor TFIIB repeat (TFIIB, PF00382), homeodomain (HOX, PF00046), fungal_trans domain (FTD, PF04082), mobility group (HMG, PF00505), and high heat shock factor (HSF, PF00447) TFs were downloaded from the InterProScan database. HMMER software was used to perform a global alignment screening of the protein sequences of the *P. portentosus* genome, with a threshold E value < 10^−5^, and manually removing short sequences with fewer than 100 amino acids. Then, candidate gene screening and conserved domain validation were conducted through the NCBI CD-search online website (https://www.ncbi.nlm.nih.gov/Structure/cdd/wrpsb.cgi; accessed on 6 February 2025). Finally, the online program MEME-suite (http://meme-suite.org/index.html; accessed on 6 February 2025) was used to identify the conserved motifs of the *P. portentosus* TF protein, and TBtools software was utilized to visualize the conserved motifs and gene structure pattern diagrams.

### 2.9. Differentially Expressed Metabolites and Enrichment Analysis

Three samples of mycelium were collected from the *P. portentosus* YAF023 under the following cultivation conditions: PDA solid (L016_PDA), BDMY liquid (L016), and BDMY liquid with added valproic acid (L016_BWS), and Shanghai Personalbio Biotechnology Co., Ltd. was employed to conduct LC-MS/MS analysis. Principal component analysis (PCA) and projections to latent structures discriminant analysis (PLS-DA) were performed on the obtained metabolomic data, using R2X, R2Y, and Q2 values to describe the quality of the model. Based on the OPLS-DA, the variable importance for the projection (VIP) values of all metabolites were obtained and then combined with the *p*-value or fold change from univariate analysis to further screen for differentially expressed metabolites (DEMs). The screening criteria for DEMs are VIP > 1, *p* < 0.05. The obtained DEMs’ information was mapped to the KEGG database to obtain the enriched KEGG metabolic pathways.

### 2.10. Transcriptome Sequencing and Differential Gene Expression Analysis

Similarly to metabolomics sequencing, samples of *P. portentosus* mycelium under different cultivation conditions were collected and sent to PacBio Technology Co., Ltd. (Menlo Park, CA, USA) for transcriptome sequencing (RNA-seq). Using NGS based on the Illumina HiSeq platform (Illumina, CA, USA) and paired-end sequencing, samples under three different cultivation conditions were sequenced. After RNA-seq, the first step was to analyze raw reads in fastq format for quality control, filtering out some adapter sequences and low-quality Reads to obtain clean reads. The Q20 (%) and Q30 (%) content of the clean reads were calculated and data quality assessment performed on the clean reads. Differentially expressed genes (DEGs) were analyzed using HISAT2 (v2.1.0) software to align the clean data to the reference genome. Based on FPKM values, DEseq2 was used for differential screening analysis. GO functional enrichment analysis and KEGG pathway enrichment analysis were performed on DEGs. The criteria for selecting DEGs were as follows: expression difference fold change |log2FoldChange| > 1, significance *p*-value < 0.05. The significantly enriched GO terms and KEGG pathways of the DEGs were identified, and their main biological functions were determined. Based on the sequence numbers of PKS, TPS, and TFs in the whole-genome data of *P. portentosus*, their gene quantification indicators (FPKM values) were searched in the transcriptomic data, and TBtools software was used to draw an interactive heatmap to analyze the expression levels of the target genes.

### 2.11. Prediction of Transcription Factor Binding Sites

Based on the whole-genome and transcriptome data of *P. portentosus*, the TBtools software was used to extract the DNA sequences of the 2000 bp upstream region of the start codon of *PpPKS* and *PpTPS* genes that have similar expression patterns to the TFs. Subsequently, the JASPAR online software was used to predict potential binding sites of TFs in the promoter regions of their co-expressed genes, with a confidence level set to 100%.

### 2.12. Quantitative Real-Time PCR Analysis

To verify the reliability of RNA-seq results and the expression levels of important genes, nine TFs, PKS, and TPS genes of *P. portentosus* under different cultivation conditions were selected for validation (*PpPKS1*, *PpHOX4*, *PpPKS2*, *PpTFIIB3*, *PpPKS5*, *PpHSF5*, *PpSTCs 6*, *PpZn-clus 9*, and *PpSTCs 8*). RNA from the various treatment groups was reverse-transcribed to obtain first-strand cDNA. Using Tubulin alpha as the reference gene, gene-specific primers were created using Primer Premier 5.0 software to assess their expression levels (Table S1). The 2^−ΔΔCt^ technique was used to calculate relative transcription levels. One-way ANOVA was used to statistically evaluate the data; the standard deviation (SD) is shown by the error bars, and significance was determined at *p* < 0.05.

## 3. Results

### 3.1. Genome Assembly

**Figure 1:**
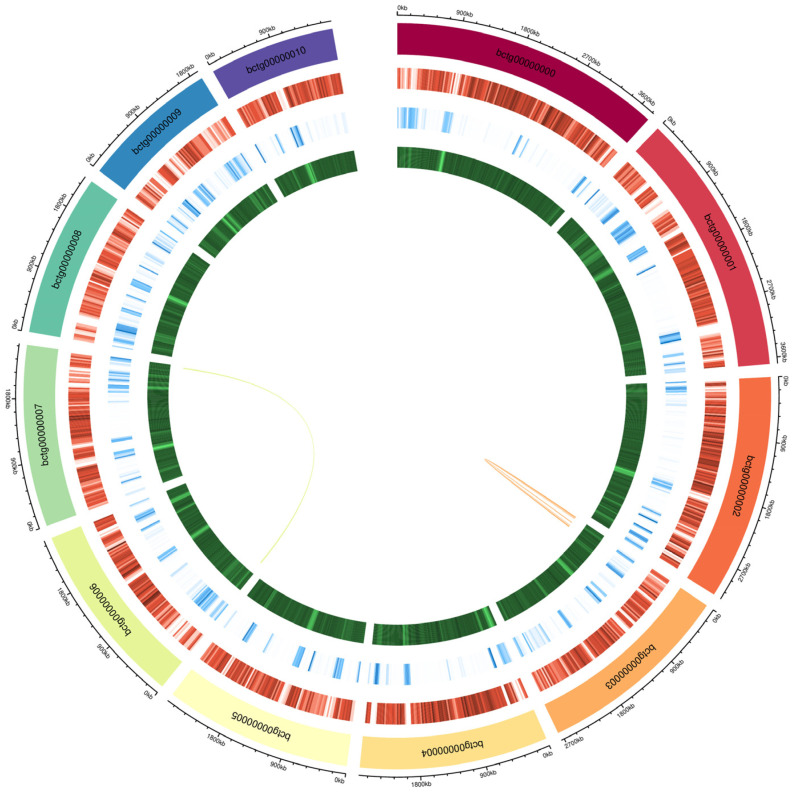
Genome diagram of *P. portentosus* YAF023 genome. From the outer to the inner circles, the layers represent: chromosome coordinate; chromosome name; gene density; genome duplication; GC content; collinear block.

The sequence length obtained from sequencing the genome of *P. portentosus* was 31,421,828 bp, which contained 15 scaffolds, with an average scaffold length of 2,094,788.53 bp, the longest scaffold being 3,801,475 bp, N50 of 2,638,669 bp, N90 of 1,761,355 bp, and a GC content of 48.91% (Figure 1). As shown in Figure 1, the genome diagram of *P. portentosus* consists of five circles. The alignment rate of NovaSeq 6000 (Illumina, USA) data reached 99.23%, and the BUSCO assessment indicated that the assembly completeness was 95.9%. The results indicate that the genome assembly quality is good. We predicted a total of 7928 protein-coding genes with an average cds sequence length of 1500.19 bp and an average of 8.39 exons per predicted gene (Table 1). The number of long terminal repeats (LTRs) was predicted to be 3,552,795 bp, which accounted for 11.31% of the whole genome, the number of DNA transposons was 890,177 bp, which accounted for 2.83% of the whole genome, and the number of satellite DNA (Satellite) repeats was 1737 bp, which accounted for 0.01% of the whole genome. For non-coding RNAs, 134 tRNAs, 4 rRNAs, and 18 snRNAb were predicted.

### 3.2. Genome Annotation

**Figure 2:**
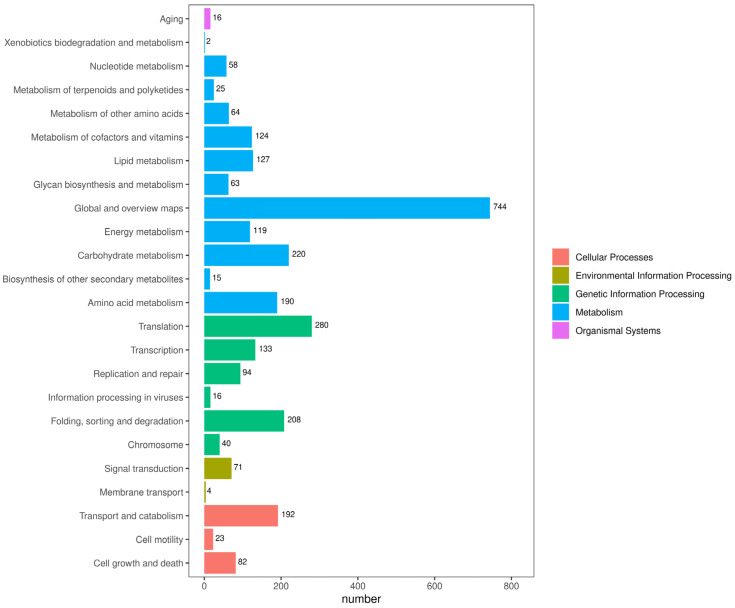
KEGG functional annotation of *P. portentosus* YAF023 genes encoding the proteins. The horizontal axis represents the number of annotated genes under each pathway category; the vertical axis represents the pathway classifications. Different colors indicate the broader categories to which they belong.

To obtain comprehensive gene function information, a similarity analysis was conducted on the 7928 non-redundant genes of *P. portentosus* in the publicly available protein databases. The highest number of functional genes was detected in *P. portentosus* YAF023 in the Nr database (7638/96.34%), followed by Uniprot annotated genes (7554/95.28%), Refseq (7398/93.31%), Interproscan (7305/92.14%) Pfam (5385/67.92%), GO (4981/62.83%), KEGG (3112/39.25%), Tigerfam (1764/22.25%), Pathway (1702/21.47%), and KOG (1625/20.50%). By comparing the annotation results with the Nr database, we counted and plotted the species distribution. The highest homology was found in *Phlebopus* sp. *FC_14*, with 6040 sequences, accounting for 84.09%; followed by *Hydnomerulius pinastri* MD-312, with 497 sequences, accounting for 6.92%; and other species with 1101 sequences, accounting for 8.99% (Figure S1). We identified 237 WD40, 140 MFS, and 138 Pkinase genes in the Pfam domains of the *P. portentosus* genome (Figure S2). According to the KEGG analysis, *P. portentosus* unigenes were mainly involved in metabolism, genetic information processing, cellular processes, and environmental information processing, organismal systems, and among the 24 subclasses, “Global and overview maps” (744) was the most enriched pathway, followed by “Translation” (280) and “Translations” (301), which were the most enriched pathways, and “Carbohydrate metabolism” (220) with a higher number of annotated genes (Figure 2). The 1,625 annotated unigenes in the KOG database were grouped into 25 categories, with the most annotated categories being “General function prediction only” (384), “Function unknown” (215), and “Signal transduction mechanisms” (189). Among them, there were 11 unigenes annotated to “Secondary metabolites biosynthesis, transport, and catabolism” (Figure S3). The results of GO functional annotation showed that 4,981 unigenes were annotated into three functional categories: biological process, cellular component, and molecular function. In terms of biological processes, genes were mainly involved in “translation” (95), “protein transport” (87), and “carbohydrate metabolic process” (74); cellular components were mainly distributed in the “integrative component of membrane” (74); genes were mainly involved in the “cellular component of membrane” (74). membrane” (1275), ‘nucleus’ (398), and ‘cytoplasm’ (247); and the molecular function classification was mainly clustered in ‘ATP binding’ (398) and ‘carbohydrate metabolic process’ (247), ‘ATP binding’ (545), and ‘metal ion binding’ (353) (Figure S4).

### 3.3. Extended Annotation

#### 3.3.1. Carbohydrate Enzyme Annotation

In the *P. portentosus* genome, 201 genes encoding CAzymes were identified. This includes 89 glycoside hydrolases (GHs), 53 glycosyltransferases (GTs), 5 polysaccharide lyases (PLs), 42 auxiliary activities (AAs), 8 carbohydrate esterases (CEs), and 4 carbohydrate-binding modules (CBMs) (Figure S5). The genomic analysis of *P. portentosus* also revealed the presence of fewer copies of CAZyme acting on cellulose, xylan, pectin, and lignin. The total number of CAZyme family genes in *P. portentosus* was consistent with the range of ECM fungi, and *P. portentosus* had the same number of family genes as *Pisolithus tinctorius* AA and a similar number of GH family genes (Table 2). The genomes of ECM fungi are larger than those of saprophytic fungi, and the average number of CAZyme family genes detected in the genomes of ECM fungi (288) was lower than those detected in saprophytic fungi (550); we observed that, except individual ECM fungi with higher numbers of GT families than those of saprophytic fungi, the number of enzymes of the AA, CBM, CE, GH, and PL families was lower than that of saprophytic fungi (Table 2).

GHs were distributed in 21 families, among which families GH13 (7 copies), GH16 (15 copies), and GH31 (7 copies) were more abundant, and AAs included 8 families, among which 3 families, AA1_1 (12 copies), AA3_2 (6 copies), and AA7 (9copies) were more abundant. GTs include 22 families, of which 14 chitin synthetases belong to the GT2 family. CEs were distributed in 4 families: CE4 (2 copies), CE9 (2 copies), CE16 (3 copies), and CE17 (1 copy). PLs were mainly distributed in the PL8, PL14 (2 copies), PL35, and PL38 families. *P. portentosus* contains 15 genes encoding laccase (AA1) and 1 gene encoding peroxidase (AA2), indicating that *P. portentosus* has the potential to decompose lignin substrates. A total of 7 cellulase genes were identified in *P. portentosus*, including 2 genes encoding exoglucanases (GH55), 1 gene encoding endo-*β*-1,4-glucanase (GH9), and 4 genes encoding *β*-glucosidases (GH3), suggesting that *P. portentosus* may have a low ability to degrade cellulose. Additionally, there are 7 genes encoding α-amylases (GH13), 2 genes encoding glucoamylases (GH15), 7 genes encoding α-glucosidases (GH31), and 4 genes encoding starch-binding proteins (2 CBM20 and 2 CBM22).

#### 3.3.2. Virulence Factor (VFDB) Analysis

The ability of pathogens to invade hosts and reproduce within them is the result of the coordinated action of a series of VFDBs. Bacterial virulence factors are typically proteins that enable pathogens to parasitize hosts, including gene products related to adhesion, invasion, secretion systems, toxins, and iron acquisition systems. These virulence factors help the causative organisms establish infection of the host, respond to the host’s immune system to survive in the host, and cause disease in the host. The genome of *P. portentosus* YAF023 was annotated to a total of 837 VFDBs in the VFDB database, which consisted of 14 categories: adherence (45), biofilm (2), effector delivery system (236), exoenzyme (5), exotoxin (76), immune modulation (154), motility (49), nutritional/metabolic factor (190), post-translational modification (3), regulation (6), stress survival (41), antimicrobial activity/competitive advantage (11), invasion (6), and others (13). VFDBs are mainly distributed across three categories: effector secretion systems (236), nutrient/metabolic factors (190), and immune regulation (154). These categories include HSI-I (66), T4SS secreted effectors (59), thioquinolobactin (33), MymA operon (33), GPL locus (30), and LPS (17), among others. Additionally, there are exotoxins such as phytotoxin syringopeptin (20) and colibactin (12), as well as motility factors like polar flagella (31). These VFDBs may play an important role in the process of bacterial adhesion, invasion of the host, and subsequent disease development.

#### 3.3.3. Pathogen–Host Interaction (PHI) Analysis

**Figure 3:**
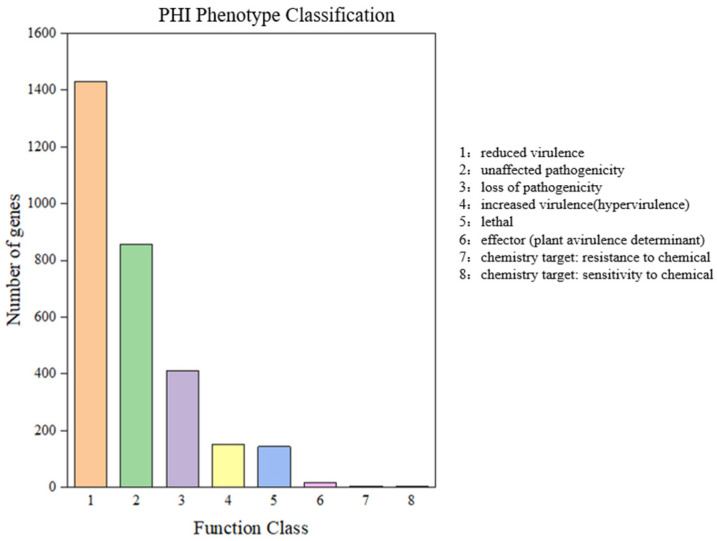
The phenotype types of annotated PHI from *P. portentosus* YAF023 genome. The horizontal axis represents the phenotype types. The vertical axis represents the number of annotated genes under each phenotype type.

The PHI database is mainly sourced from fungi, oomycetes, and bacterial pathogen infections in hosts including animals, plants, fungi, and insects. PHI analysis of *P. portentosus* identified 3334 genes that were annotated, of which the most abundant was “reduced virulence” with 1431 genes, followed by “unaffected pathogenicity” (856), “loss of pathogenicity” (412), “increased virulence (unaffected pathogenicity)” (856), “loss of pathogenicity” (412), “increased virulence (hypervirulence)” (153), “lethal” (144), “effector (plant avirulence determinant)“ (16), “chemistry target: resistance to chemical” (5), and “chemistry target: sensitivity to chemical” (5) (Figure 3). The results indicate that the main annotated genes are “reduced virulence” genes, while pathogenicity is not affected, suggesting that *P. portentosus* is not a highly pathogenic fungi.

#### 3.3.4. Cytochrome P450 Annotation

**Figure 4:**
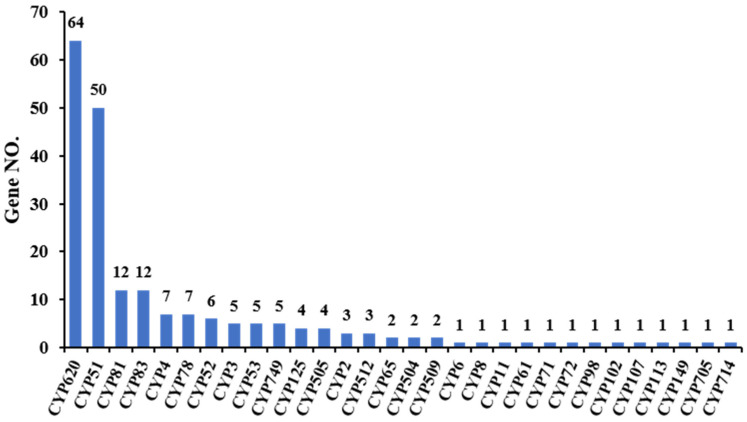
CYP450 annotation of *P. portentosus* YAF023 genome. The horizontal axis represents the CYP450 types. The vertical axis represents the number of annotated genes under each CYP450 type.

Cytochrome P450 (CYP450) is a large family of proteins with ferrous heme as a cofactor that catalyzes the oxidation of many kinds of substrates, and it is involved in the metabolism of endogenous and exogenous substances, including drugs and environmental compounds. The CYP540 superfamily currently comprises about 9,000 proteins forming more than 800 families. They provide biodefense (detoxification of exogenous substances, antibiotic production) and participate in the biosynthesis of important endogenous molecules, especially steroids [41]. Based on these two roles, CYP51 is a lanosterol 14-alpha demethylase involved in the 14-demethylation of sterol precursors, and this demethylation step is common throughout all organisms; the CYP53 family is present in many saprophytic species as well as in many basidiomycete fungi, which can degrade phenol derivatives as a carbon source [42]. Based on the CYP450 database, 206 putative CYP450 genes, including enzymes in 30 superfamilies, were identified from the whole genome of *P. portentosus*. The CYP620 superfamily has the highest number of genes (64), followed by CYP51 (50), CYP81 (12), and CYP83 (12) (Figure 4).

### 3.4. Metabolome Analysis of Different Cultivation Conditions

**Figure 5:**
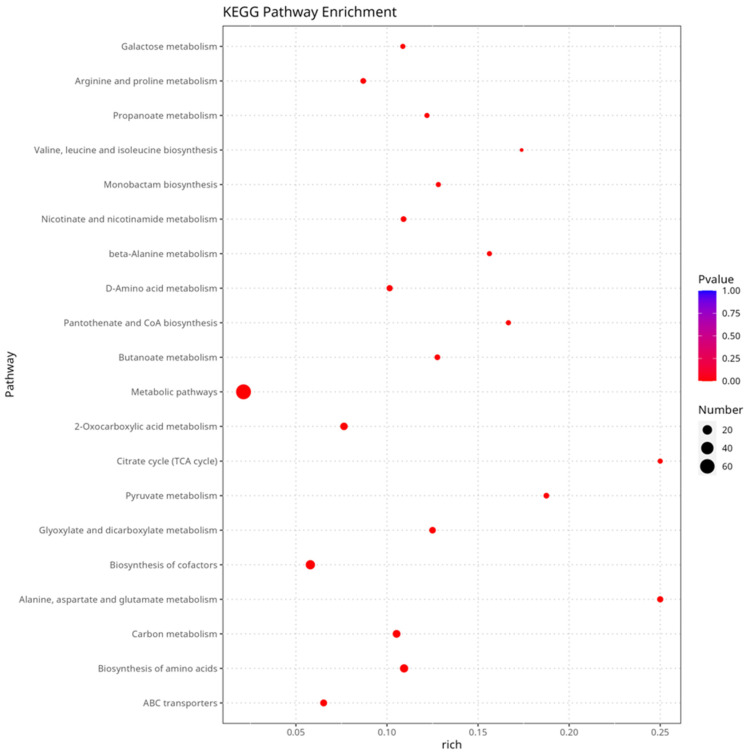
KEGG pathway of differential metabolite enrichment of *P. portentosus* cultured in L016 and L016_BWS medium. The horizontal axis represents −log10 (*p*-value). The vertical axis represents the KEGG pathway.

The metabolites were identified through LC/MS to determine the chemical composition profile of *P. portentosus* under different cultivation conditions. A total of 1582 metabolites were identified, and the numbers of positive and negative ion modes were 933 and 649, respectively. This mainly includes organoheterocyclic compounds, organic acids and derivatives, lipids and lipid-like molecules, benzenoids, organic oxygen compounds, phenylpropanoids and polyketides, organic nitrogen compounds, nucleosides, nucleotides, and analogs, alkaloids, and derivatives (Table 3). In the PCA plot, the QC samples were grouped and the three groups were differentiated, with parallel samples within the groups being close in composition, suggesting that they had similar metabolic profiles and that the overall analysis was reliable and reproducible. Biological replicates for each of the three different cultures clustered in different regions, indicating significant differences in metabolites (Figure S6). L016 vs. L016_BWS had 651 DEMs (346 upregulated, 305 downregulated). L016_PDA vs. L016 had 574 DEMs (259 upregulated, 315 downregulated). L016_PDA vs. L016 had 607 types of DEMs (276 upregulated, 331 downregulated) (Figure S7a,b). To gain a deeper understanding of the metabolic pathways involved in DEMs, KEGG significance enrichment analysis was performed on DEMs, and 948 DEMs were identified, involving a total of 84 metabolic pathways. The top 20 KEGG pathways were selected with the smallest FDR values, which are the most significantly enriched, to display the enrichment factor graph. The KEGG metabolic pathways such as ABC transporters, biosynthesis of amino acids, 2-Oxocarboxylic acid metabolism, biosynthesis of cofactors, and arginine and proline metabolism are significantly enriched (Figure 5).

### 3.5. Transcriptome Analysis of Different Cultivation Conditions

**Figure 6:**
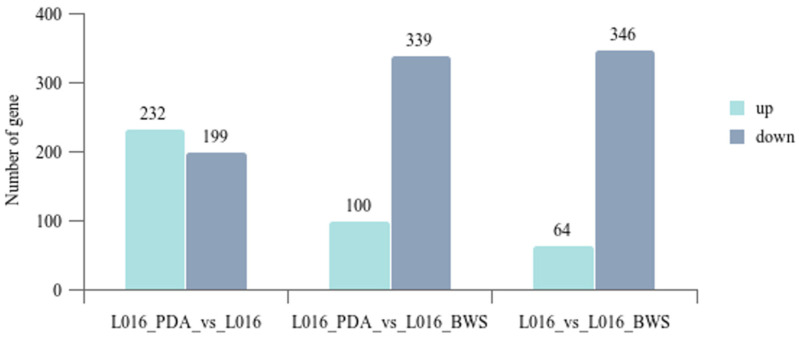
DEGs analyses of *P. portentosus* in different cultivation conditions. The horizontal axis represents different cultivation conditions. The vertical axis represents the number of DEGs.

Transcriptome analysis of *P. portentosus* YAF023 under different cultivation conditions showed that the clean reads for L016_PDA, L016, and L016_BWS were 98.87%, 98.96%, and 98.82%, respectively, and the read mapping rates for the sequencing samples (Total Mapped) were 96.96%, 95.35%, and 96.24%, respectively. L016_PDA vs. L016 had a total of 431 DEGs, of which 232 DEGs were significantly upregulated and 199 were significantly downregulated. L016_PDA vs. L016 had a total of 439 DEGs, of which 100 DEGs were significantly upregulated and 339 downregulated. L016 vs. L016_BWS had 410 DEGs, 64 DEGs upregulated and 346 downregulated (Figure 6). KEGG pathway enrichment analysis indicates that DEGs are mainly enriched in pathways such as aflatoxin biosynthesis, phenylalanine, tyrosine and tryptophan biosynthesis, ribosome, MAPK signaling pathway—yeast, and glutathione metabolism. To understand the functions of DEGs in related biological processes, GO function enrichment analysis indicates that DEGs are mainly enriched in molecular function (MF), cellular component (CC), and biological process (BP). The results showed that DEGs were mainly enriched in functions related to four-way junction helicase activity in MF, cytosolic ribosome in CC, and antibiotic biosynthetic process in BP.

### 3.6. Analysis of Biosynthetic Gene Clusters

The whole-genome sequence of *P. portentosus* was submitted to the antiSMASH database for SM BGC analysis. AntiSMASH analysis showed that *P. portentosus* has 24 BGCs, including 18 TPS, 1 NRPS, and 5 PKS. The five PKS genes of *P. portentosus* include one NR-PKS: *PpPKS1* (SAT-KS-AT-PT-ACP-TE), one PR-PKS: *PpPKS2* (SAT-KS-AT-DH-KR-ACP), two HR-PKS: *PpPKS3*, *PpPKS4* (KS-AT-DH-MT-ER-KR-ACP-TE), and one hybrid PKS-NRPS: *PpPKS5* (KS-AT-DH-ER-KR-ACP-C).

#### 3.6.1. Ethyl Orsellinate Biosynthesis Gene in *P. portentosus*

**Figure 7:**
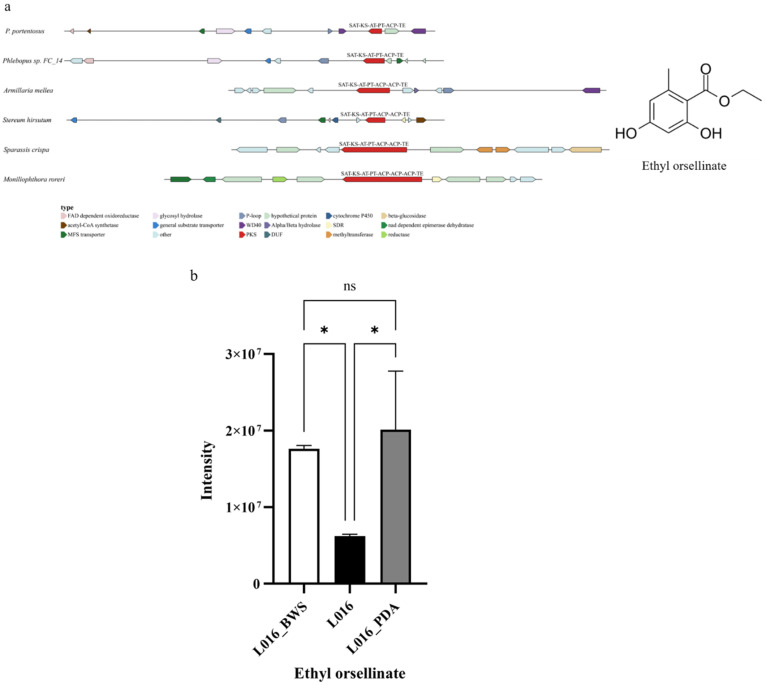

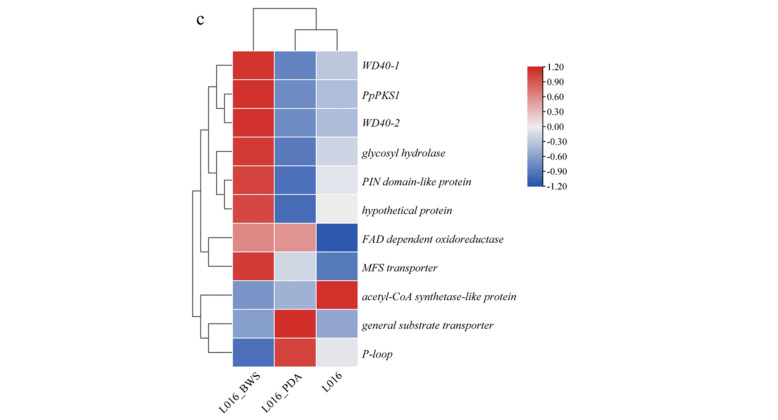
The biosynthesis of Ethyl orsellinate: (**a**) comparison of biosynthesis of putative Ethyl orsellinate biosynthetic gene cluster; (**b**) relative abundance of Ethyl orsellinate under different cultivation conditions. The horizontal axis represents different cultivation conditions. The vertical axis represents the intensity of Ethyl orsellinate. The values are shown as mean ± SD (n = 3). * *p* < 0.05. ns: no significant difference. (**c**) Transcriptional expression of PpPKS1 and surrounding genes under different cultivation conditions. The horizontal axis represents different cultivation conditions. The vertical axis represents gene expression. Expression levels are color-coded, with red and blue representing high and low expression, respectively.

The PpPKS1 domain SAT-KS-AT-PT-ACP-TE is similar to the PKS domains related to orsellinic acid synthesis in Phlebopus sp. FC_14, Armillaria mellea, Stereum hirsutum, Sparassis crispa, and Moniliophthora roreri, and their protein sequences were clustered together. Therefore, it is speculated that the product of PpPKS1 is the orsellinic acid derivative Ethyl orsellinate (Figure S8). Additionally, the analysis of this PKS and its surrounding genes in the other five genomes showed results similar to those of PpPKS1. The NR-PKS gene clusters in the Phlebopus fungi all contain FAD-dependent oxidoreductase, MFS transporter, glycosyl hydrolase, general substrate transporter, and P-loop. At the same time, horizontal gene transfer and gene deletion phenomena were observed in each gene cluster. *A. mellea* has an extra Alpha/Beta hydrolase gene in its modifier genes. *S. hirsutum* has an extra cytochrome P450, a short-chain dehydrogenase/reductase (SDR) gene. *S. crispa* has an additional beta-glucosidase. The modified genes of M. roreri contain beta-glucosidase, nad-dependent epimerase dehydratase, reductase, and SDR genes (Figure 7a). Different species exhibit a high degree of conservation of core PKS genes and a remarkable diversity of surrounding modifier genes in Ethyl orsellinate synthesis, revealing the coexistence of a high degree of conservatism and diversity in the biosynthetic pathway. Metabolome analysis showed that the content of the target compound Ethyl orsellinate was 2.84 times higher than that in L016 after induction with the addition of valproic acid under liquid culture conditions (Figure 7b). Transcriptome analysis showed that the expression of the PpPKS1 gene, which is involved in the synthesis of Ethyl orsellinate, was significantly upregulated in L016_BWS, and its expression was 2.16-fold higher than that of L016; the genes surrounding this PKS also showed high expression levels in L016_BWS (Figure 7c). It is worth noting that although Ethyl orsellinate was the most abundant in L016_PDA, it had a large error line, probably due to uneven sampling leading to this result.

#### 3.6.2. 6-Methylsalicylic Acid Biosynthesis Gene in *P. portentosus*

**Figure 8:**
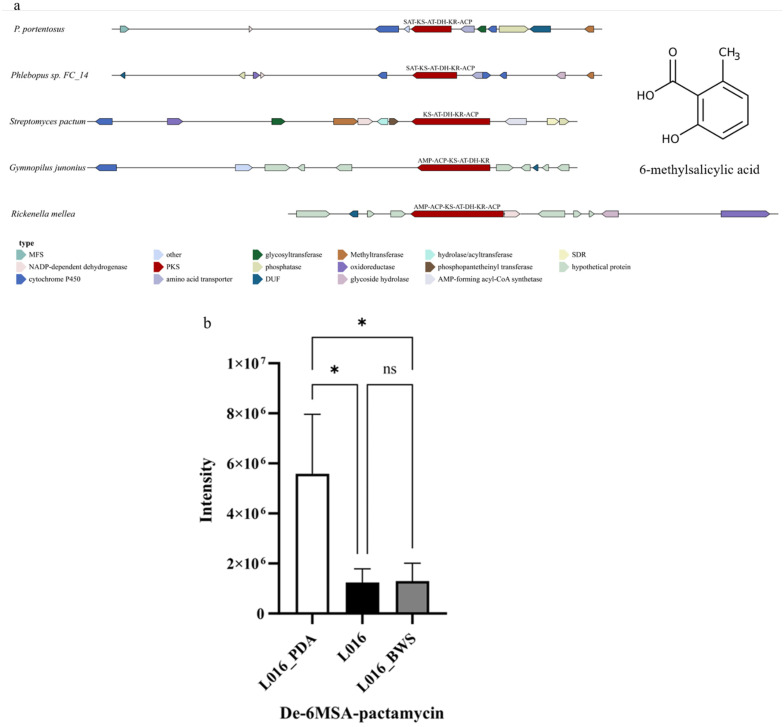

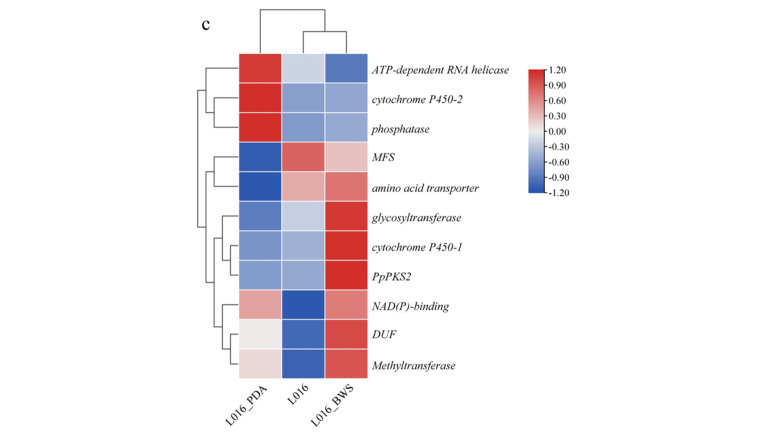
The biosynthesis of the 6-methylsalicylyl acid: (**a**) comparison of biosynthesis of the 6-methylsalicylyl acid biosynthetic gene cluster; (**b**) relative abundance of De-6MSA-pactamycin under different cultivation conditions. The horizontal axis represents different cultivation conditions. The vertical axis represents the intensity of De-6MSA-pactamycin. The values are shown as mean ± SD (n = 3). * *p* < 0.05, ns: no significant difference. (**c**) Transcriptional expression of PpPKS2 and surrounding genes under different cultivation conditions. The horizontal axis represents different cultivation conditions. The vertical axis represents gene expression. Expression levels are color-coded, with red and blue representing high and low expression, respectively.

Clustering PpPKS2 (SAT-KS-AT-DH-KR-ACP) with three PKS genes related to the synthesis of 6-Methylsalicylic acid downloaded from NCBI, which were clustered in a single clade and had similar structural domains (Figure S9), it was hypothesized that PpPKS2 could catalyze the synthesis of 6-Methylsalicylic acid or its derivatives. PpPKS2 and the other four fungal PKSs all contain DUF genes, NADP-dependent dehydrogenase (except Gymnopilus junonius), and cytochrome P450 (except Rickenella mellea); PpPKS2, P. sp. FC_14, and Streptomyces pactum all contain phosphatase and methyltransferase genes, and PpPKS2 and S. pactum contain glycosyltransferase genes (Figure 8a). The content of De-6MSA-pactamycin in L016_BWS was higher than that in L016 under liquid cultivation conditions (Figure 8b), and the expression of the PpPKS2 gene was higher than that of the cultured ones without an inducer after the addition of valproic acid inducer in the transcriptome, suggesting that the addition of valproic acid inducer to *P. portentosus* has a certain effect on 6-Methylsalicylic acid (Figure 8c).

#### 3.6.3. Cytochalasin Z5 Biosythesis Gene in *P. portentosus*

**Figure 9:**
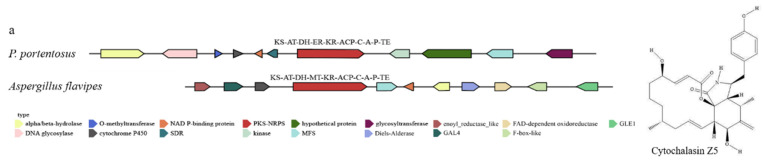

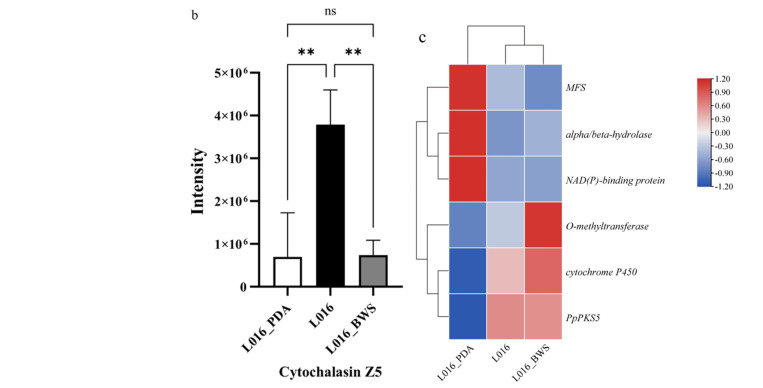
The biosynthesis of Cytochalasin Z5: (**a**) comparison of biosynthesis of Cytochalasin Z5 biosynthetic gene cluster; (**b**) relative abundance of Cytochalasin Z5 under different cultivation conditions. The horizontal axis represents different cultivation conditions. The vertical axis represents the intensity of Cytochalasin Z5. The values are shown as mean ± SD (n = 3). ** *p* < 0.01. ns: no significant difference. (**c**) Transcriptional expression of PpPKS5 and surrounding genes under different cultivation conditions. The horizontal axis represents different cultivation conditions. The vertical axis represents gene expression. Expression levels are color-coded, with red and blue representing high and low expression, respectively.

PpPKS5 (KS-AT-DH-ER-KR-ACP-C-A-P-TE) clusters with the PKS-NRPS of A. flavipes and shows high homology (Figure S10). The PKS-NRPS gene product of A. flavipes is cytochalasan. Cytochalasin Z5 was detected in the metabolomics data of *P. portentosus*, and its biosynthetic gene cluster includes PKS-NRPS, trans-ER, α/β-hydrolase-related Cytochalasins scaffold genes, as well as modification genes such as cytochrome P450, MFS, and O-methyltransferase. Therefore, it is speculated that *P. portentosus* can synthesize Cytochalasin Z5 (Figure 9a). Metabolome analysis showed that the content of Cytochalasin Z5 in L016 was significantly higher than in L016_PDA and L016_BWS cultures (Figure 9b). Transcriptome analysis indicated that the expression levels of the PpPKS5 gene, which synthesizes Cytochalasin Z5, and the surrounding modification gene cytochrome P450 were significantly upregulated in L016 compared to L016_PDA (Figure 9c).

### 3.7. Characterization of PpTPS Proteins

**Figure 10:**
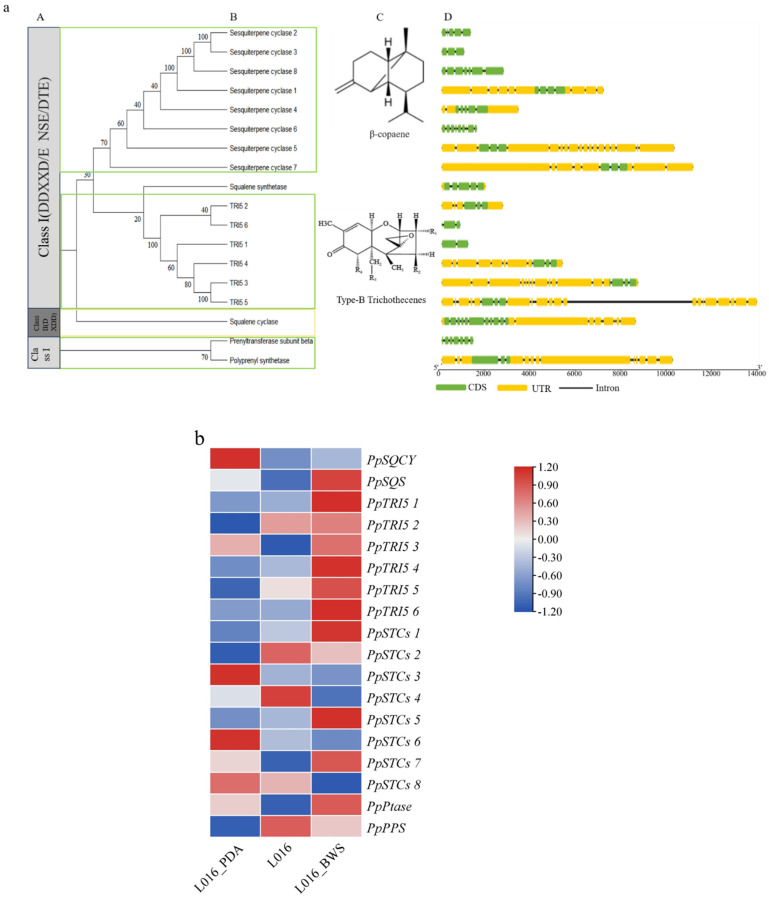
Genome-wide identification of TPS and gene expression analysis in *P. portentosus*. (**a**) Phylogenetic analysis and classification of the TPS gene family and analysis of TPS gene structure and motif: (**A**) enzyme type and motif; (**B**) phylogenetic analysis; (**C**) related compounds; (**D**) TPS genes structures. CDS: sequence of coding; UTR: untranslated region; lines indicate introns. (**b**) TPS gene expression of *P. portentosus* under different cultivation conditions. The horizontal axis represents different cultivation conditions. The vertical axis represents gene expression. Expression levels are color-coded, with red and blue representing high and low expression, respectively.

From the *P. portentosus* genome, 18 TPS genes were found, including 6 TRI5, 8 STCs, 1 PPS, 1 Ptase, 1 SQS, and 1 SQCY (Figure 10a(B)), among which *PpSQCY* belongs to the Class II, and its protein has the motif “DXDD”. *PpSQCY* belongs to Class II and its protein has the motif “DXDD”, while the rest of *PpTPS* belongs to Class I and has the highly conserved aspartic acid-rich motif “DDXXXD” (Figure 10a(A)). The expression levels of these genes differed significantly at different stages, determining differences in terpene product synthesis (Figure 10b). The expression levels of *PpTRI5 1–6*, *PpPtase*, *PpSTCs 1*, *5*, and *7*, and *PpSQS* genes were significantly higher in L016_BWS than in L016 and L016_PDA cultivation conditions. In L016 liquid culture, *PpSTCs 2*, *4*, and *PpPPS* gene expression was more active than in L016_PDA and L016_BWS, which may be related to specific biological processes or metabolic pathways in the liquid environment. In solid culture conditions (L016_PDA), *PpSQCY*, *PpSTCs 3*, *6*, and *8* gene expression was more active than in liquid culture (Figure 10b). The *PpTRI5* protein sequence clusters with the TRI5 protein sequences of *Beauveria bassiana* (XP_008596951), *Trichoderma reesei* (XP_006964535), and *Trametes versicolor* (XP_008037460), according to phylogenetic analysis (Figure S9). The *PpTRI5* genes encode trichodiene synthases, which are responsible for producing tetracyclic sesquiterpene B-type trichodiene (Figure 10a(C)). *PpSQS* and the squalene synthase of *Lentinula edodes* (GAW09328) cluster together, with 100% homology. *PpPPS* has high homology with the prenyl synthases of *Trametes maxima* (KAI0673618) and *Trametes meyenii* (KAI0652351). *PpPtase* clusters with the Ptases of *S. crispa* (XP_027616833) and Grifola frondosa (OBZ72750), encoding isoprenyl transferases. *PpSTCs* showed high homology with the sesquiterpene synthases of *Coniophora puteana* (XP_007771895; XP_007772164) and *Lignosus rhinocerus* (KX281944), and it was hypothesized that they might be involved in the synthesis of *β*-copaene, δ-cadinol, and α muurolene analogs or derivatives (Figure 10a(C)) [43]. Then, we analyzed the exon/intron boundaries of the *PpTPS* gene, which can provide additional evidence for the evolution of multiple gene families [44]. The results show that, except for *PpPtase*, *PpSTCs6*, and *PpSTCs8*, which have 6, 8, and 7 introns, respectively, most Class I TPS genes contain 2-4 introns in the CDS region (Figure 10a(D)). Additionally, the Class II TPS gene *PpSQCY* has 10 introns.

### 3.8. Analysis of Lanosterin Transcriptome and Metabolome Expression Under Different Cultivation Conditions

**Figure 11:**
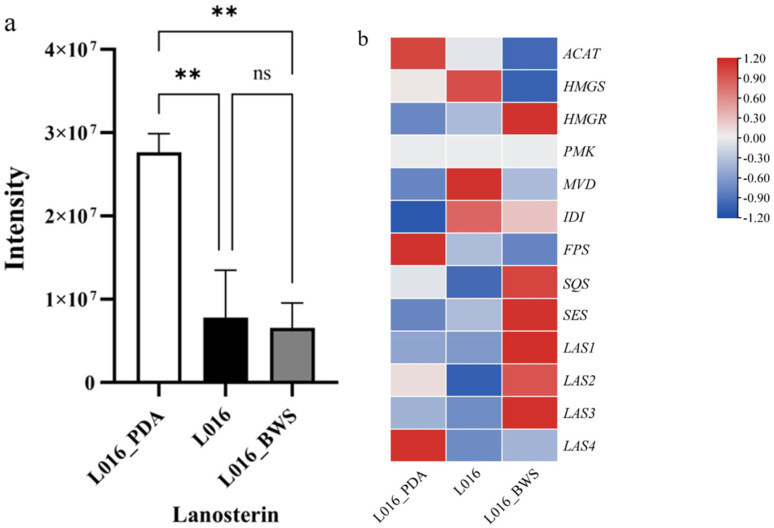
Lanosterol content under different culture conditions and expression of its biosynthetic genes. (**a**) Relative abundance of lanosterin under different cultivation conditions. The horizontal axis represents different cultivation conditions. The vertical axis represents the intensity of lanosterin. ** *p* < 0.01. ns: no significant difference. (**b**) Expression of genes related with lanosterin biosynthesis. The horizontal axis represents different cultivation conditions. The vertical axis represents gene expression. Expression levels are color-coded, with red and blue representing high and low expression, respectively.

In the metabolomic analysis, the compound Lanosterin (LAS) was detected, and we observed that the LAS content in L016_PDA was significantly higher than under liquid culture conditions (L016 and L016_BWS) (Figure 11a). In the genome and transcriptome, the genes involved in its biosynthetic pathway, Acetyl-CoA acetyltransferase B (ACAT), Hydroxymethylglutaryl-CoA synthase (HMGS), 3-hydroxy-3-methylglutaryl-coenzyme A reductase (HMGR), Phosphomevalonate kinase (PMK), Diphosphomevalonate decarboxylase (MVD), Isopentenyl-diphosphate delta-isomerase (IDI), Farnesyl pyrophosphate synthase (FPS), squalene synthase (SQS), squalene epoxidase erg1 (SES), and Lanosterol synthase (LAS), were identified. Based on their FPKM values, a gene expression heatmap was drawn. The analysis showed that LAS4 in L016_PDA exhibited a higher expression level compared to liquid culture conditions, and its upstream genes ACAT and FPS also showed a significant upregulation in L016_PDA (Figure 11b)**.**

### 3.9. Analysis of Gene Expression and Prediction of Binding Sites

**Figure 12:**
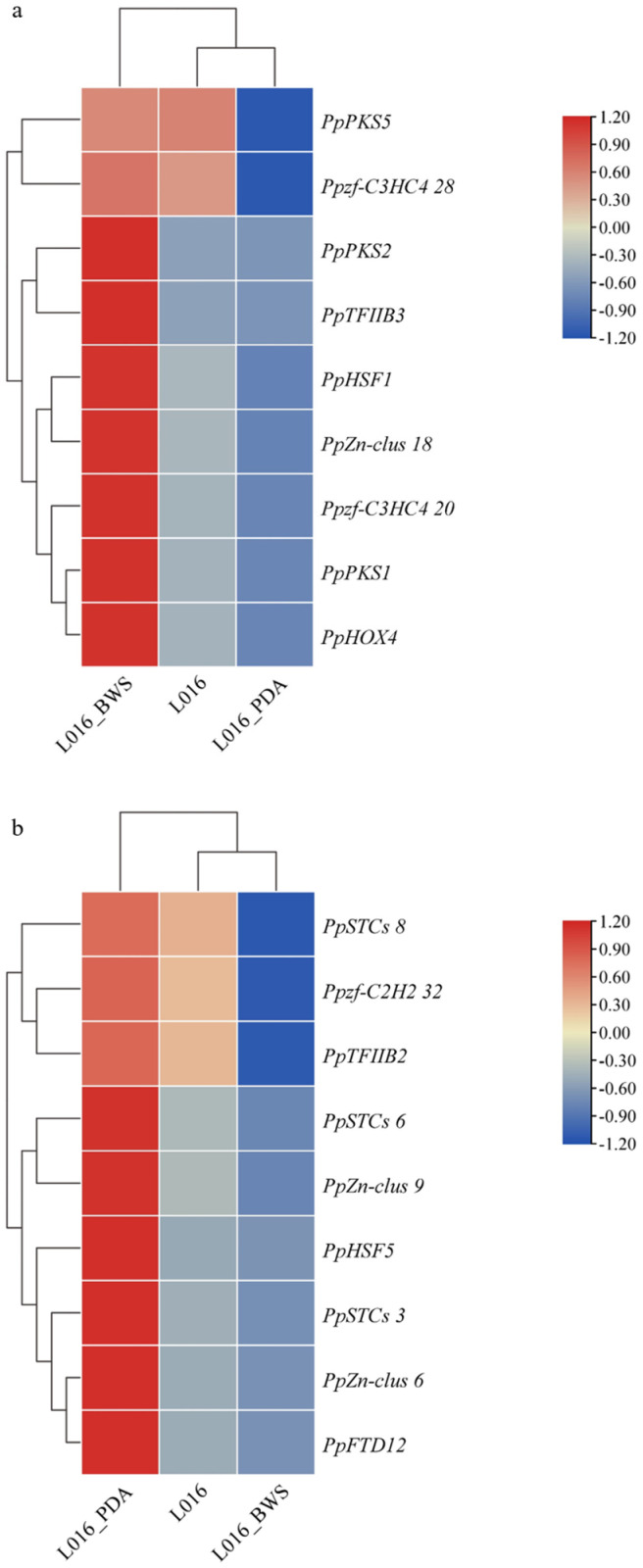
Gene expression heatmap of PpPKSs, PpTPSs, and TF gene expression interaction under different cultivation conditions: (**a**) heatmap of PKS and related TF expression; (**b**) heatmap of TPS and related TF expression. The horizontal axis represents different cultivation conditions. The vertical axis represents gene expression. Expression levels are color-coded, with red and blue representing high and low expression, respectively.

A total of 186 TF sequences were identified in the *P. portentosus* genome: MYB (13), bZIP (8), bHLH (7), zf-C2H2 (32), zf-C3HC4 (31), zf-CCCH (14), Zn-clus (34), TFIIB (4), HOX (7), FTD (22), HMG (8), and HSF (6), named PpMYB1-PpHSF6. Using TBtools to visualize its phylogenetic tree, conserved motifs, and conserved domains, members of the same subgroup with similar evolutionary relationships exhibited high similarity in conserved motif composition, indicating that members of the same subgroup also have high similarity in gene function. The conserved motifs of each transcription family protein differ in type, quantity, and distribution. There are variations in the composition and distribution of motifs between different subgroups, and the number of different types is consistent with the results of phylogenetic analysis. It is speculated that the different types of variations are related to multiple specific functions within their families (Figure S11). TBtools software was used to draw interactive heatmaps of PKS, TPS, and TF gene expression, visually reflecting the differences in gene expression under different cultivation conditions (Figure 12). The analysis results showed that under liquid culture conditions (L016 and L016_BWS), the expression levels of *PpPKS5* and the TF *Ppzf-C3HC4 28* genes were similar and both significantly upregulated. The expression levels of *PpPKS2* and *TFIIB3*, as well as *PpPKS1* and the TFs *PpHOX4*, *Ppzf-C3HC4 20*, *PpZn-clus 18*, and *PpHSF1* were similar, significantly upregulated in L016_BWS and significantly downregulated in solid culture L016_PDA (Figure 12a). *PpSTCs 8* and the TFs *Ppzf-C2H2 32* and *PpTFIIB2*, *PpSTCs 6* and the TF *PpZn-clus 9*, *PpSTCs 3* and the TFs *PpHSF5*, *PpZn-clus 6*, and *PpFTD12* have similar expression levels, significantly upregulated in L016_PDA and significantly downregulated in L016_BWS (Figure 12b). Based on these results, it was hypothesized that these TFs may have regulatory roles for the *PpPKS* and *PpTPS* genes. To test this hypothesis, the promoter regions (2000 bp) of the *PpPKS* and *PpTPS* genes were identified based on the whole-genome data of *P. portentosus* using TBtools software. Using the JASPAR database, we predicted the TF target binding sites in the promoter regions of the *PpPKS* and *PpTPS* genes and found several potential binding sites with high relative scores. These sites may be involved in the regulation of the *PpPKS* and *PpTPS* genes (Table 4). The *PpHSF1* binding site is “TAAT” and the PpHOX4 binding site is “ATGGAAC”, both of which are highly compatible with the promoter region of the *PpPKS1* gene, suggesting that their sites may be related to the functional regulation of the *PpPKS1* gene. The two sites were highly matched with the promoter region of the *PpPKS1* gene, indicating that their sites may be related to the regulation of *PpPKS1* gene function. The binding site of *Ppzf-C2H2 32* is “CCCCAC”, which shows a high degree of match with the promoter of the *PpSTCs 8* gene. The binding site of *PpHSF5* is “GGCC”, which highly matches the promoter region of the *PpSTCs 3* gene. These TFs may bind to the DNA sequence of the corresponding *PpTPS* gene promoter region, thereby activating the expression of the *PpTPS* gene.

### 3.10. Gene Expression Analysis by qPCR

**Figure 13:**
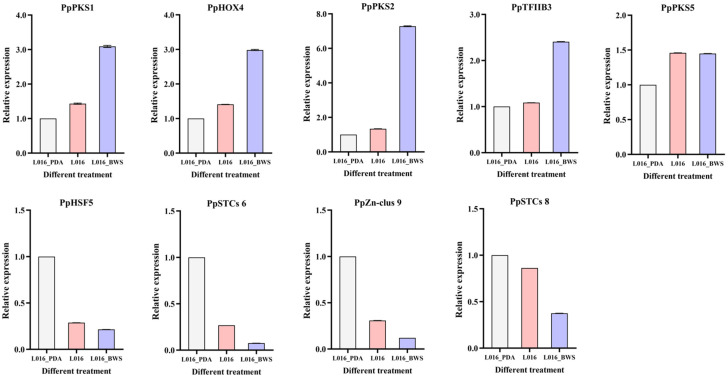
Relative expression of differentially expressed genes by qRT-PCR. The horizontal axis represents different cultivation conditions. The vertical axis represents gene expression. Bars represent the mean ± standard deviation of three biological replicates. The bar graph shows gene expression levels under different treatments.

To confirm the accuracy of the transcriptome data, we selected nine genes related to SM in *P. portentosus* for quantitative real-time PCR (qRT-PCR), including TFs, PKS and TPS genes (*PpPKS1*, *PpHOX4*, *PpPKS2*, *PpTFIIB3*, *PpPKS5*, *PpHSF5*, *PpSTCs 6*, *PpZn-clus 9*, and *PpSTCs 8*) (Figure 13). The results indicate that the expression patterns observed through qRT-PCR are consistent with the trends observed in the RNA-seq data, demonstrating the reliability of transcriptomic analysis. The expression levels of *PpPKS1*, *PpPKS2*, and their transcription factors *PpHOX4* and *PpTFIIB3* were significantly increased in L016_BWS cultivation conditions, and the expression levels of *PpSTCs 6*, *PpSTCs 8*, and their TFs *PpHSF5* and *PpZn-clus 9* were significantly increased in L016_PDA. These findings further validate that different cultivation conditions significantly affected the expression patterns of genes related to SM synthesis in *P. portentosus*. Different cultivation conditions may promote the activation of specific metabolic pathways and, thus, the synthesis of various metabolites.

## 4. Discussion

ECM fungi are mostly symbiotic with woody plants, and both plants and fungi benefit from each other. In temperate regions, ECM is associated with many plant species, including spruce, pine, and fir [36]. *P. portentosus* could form mycorrhizal-like structures in *P. yunnanensis* 1 year after inoculation with fungal mycelium [45]. Moreover, it can grow in the form of fruiting bodies without a host plant, and this method can still produce fruiting bodies two years after being initially isolated from the tissue [46]. Additionally, after inoculating *P. portentosus* into the roots of *Pinus kesiya var. langbianensis* for one year, they observed mycelial sheaths and Hartig net structures and noted the formation of dichotomous branching at the tips of lateral roots, all of which are typical structures of ECM formation in the *Pinus* genus [45]. *P. portentosus* is the first ECM fungus in the Boletaceae family to achieve industrial large-scale cultivation. In China and Thailand, *P. portentosus* has been successfully realized in greenhouses or factories which artificially cultivate and produce sporocarps in artificial substances in vitro, which has attracted much attention in the world [9]. Currently, research on *P. portentosus* mainly focuses on artificial cultivation. Although there have been studies on the SMs of *P. portentosus* under fermentation conditions [16], there have been no reports on studying the possible SMs of *P. portentosus* from the genomic, metabolomic, and transcriptomic levels. In this study, we utilized NGS+ONT sequencing technology to complete the genome sequencing of *P. portentosus* YAF023, identifying a large number of CAZyme family genes and SM biosynthesis genes. We then employed untargeted metabolomics and transcriptomics to analyze the changes in SMs and gene expression of *P. portentosus* under different cultivation conditions. Through multi-omics joint analysis, we explored the regulatory mechanisms of SMs in *P. portentosus* under different cultivation conditions.

In terms of carbon source utilization, ECM fungi and saprotrophic fungi have significant differences. ECM fungi primarily rely on carbon sources provided by host trees and have very limited ability to utilize lignocellulose in the environment, whereas saprotrophic fungi are the opposite. As a result, the genomes of ECM fungi often lack most lignocellulose-degrading enzyme genes or only retain very low copy numbers [47,48,49]. The CAZyme system in the *P. portentosus* genome is more similar to that of ECM fungi, having also lost a series of key genes for lignocellulose degradation, such as GH6, GH11, GH54, GH62, GH67, AA3_1, AA8, and AA16, which are often regarded as the signature genes for the differences between the ECM fungi and the saprophytic fungi [36]. Lignin is a complex polymer embedded in cellulose and hemicellulose to enhance the structure of plant cell walls. Laccases and peroxidases are the main enzyme families involved in lignin degradation [50]. Laccase belongs to the blue copper oxidase family, which is one of the important enzymes for lignin degradation, and can catalyze the oxidation of various phenolic compounds of lignin, oxidize the phenolic hydroxyl group into phenyl hydroxyl radical, and reduce the oxygen molecule into water; laccase is also able to degrade nonphenolic compounds in lignin in the presence of redox mediators and not only participates in the degradation of lignin, but can also regulate the formation of pigments as well as morphogenesis of the fruiting body [51]. The AA1_1 enzymes are multicopper oxidases, the most abundant family in the functional class of fungal *P. portentosus* AAs, the AA7 gene encoding gluco- or chito-oligosaccharide oxidases, and GH16 acting on xyloglucan and chitin; these genes are abundant in mycorrhizal and saprophytic fungi. AA3 belongs to the glucose–methanol–choline (GMC) oxidoreductases family, with AA3_2 including aryl alcohol oxidoreductases (AAOs) and glucose 1-oxidase (GOX). AAO, secreted by basidiomycetes, is considered to play a crucial role in lignin degradation. *P. portentosus* contains 15 genes encoding the laccase enzyme, suggesting that *P. portentosus* has the potential to break down lignin matrices; cellulose is one of the three most abundant polysaccharides in the plant cell wall, and has the basic structure of a *β*-1,4-linked D-glucose molecule [52]. Cellulose degradation involves the activities of endocellulases, exoglucanases, cellobiohydrolases, and *β*-glucosidases [53]. GH3 enzymes are widely distributed in bacteria, fungi, and plants, and they play roles in cellulose biomass degradation, remodeling of plant and bacterial cell walls, energy metabolism, and pathogen defense. In the fungus *P. portentosus*, there is no specific enzyme for hydrolyzing 1,4-beta-D-glucosidic linkages known as 1,4-*β*-cellobiosidase. *P. portentosus* contains seven cellulase genes, including two genes encoding exoglucanases (GH55), one gene encoding endo-*β*-1,4-glucanase (GH9), and four genes encoding β-glucosidases (GH3). This suggests that *P. portentosus* may have a low ability to degrade cellulose; starch is a polymer of D-glucose linked by α-1,4 bonds, synthesized by plants as a means of energy storage [54]. The GH13 family are enzymes associated with starch degradation, and GH13 and GH31 are the two main families of glycoside hydrolases, which play important roles in primary metabolism, catabolism, and glycoprotein processing. CBMs include two families, CBM20 and CBM21, which primarily provide glucoamylase and α-amylase with the ability to bind to raw starch (starch granules), and CBM20 from *A. niger* glucoamylase has been shown to not only bind to starch but also disrupt its surface, thus increasing starch decomposition rates [55]. *P. portentosus* has seven GH13 genes encoding α-amylase, two GH15 genes encoding glucoamylases, seven GH31 genes encoding α-glucosidases, and four genes encoding starch-binding (2 CBM20 and 2 CBM21).

Metabolomics technology can comprehensively analyze and identify the metabolic components within organisms, revealing the changes in metabolites within mycelium under different cultivation conditions. In this study, a total of 1582 metabolites were detected from L016, L016_BWS, and L016_PDA. The metabolites were mainly significantly enriched in four supercategories: organoheterocyclic compounds, organic acids and derivatives, lipids and lipid-like molecules, and benzenoids. This result is similar to the metabolomics of different fermentation times of *Ophiocordyceps sinensis* [56]. And the DEMs of metabolomics were mainly enriched in the pathways such as ABC transporters and amino acid metabolism, with key differential metabolites being Glycine, L-Serine, and L-Aspartic acid between *Boletus brunneissimus*, and *Leccinum extremiorientale* [57]. Amino acids are the basic components of proteins and peptides, and they are precursors for the synthesis of various SMs [58]. In fungi, the absence of amino acids may lead to metabolic imbalances [59]. A total of 948 DEMs were identified in this study, which were mainly enriched in metabolic pathways such as ABC transporters and biosynthesis of amino acids. L-Glutamine, an important substance in amino acids, has been proven to be effective in relieving stress in rats [60]. L-Glutamine was significantly downregulated in L016 vs. L016_BWS and significantly upregulated in L016_PDA vs. L016 and L016_PDA vs. L016

Correlation analysis of the metabolome and transcriptome showed that DEMs and DEGs of *P. portentosus* were mainly enriched in the pathways related to glutathione metabolism, phenylalanine metabolism, tryptophan metabolism, carbohydrate metabolism, and biosynthesis of various SMs and other related pathways. Glutathione is an essential dietary supplement with antioxidant, detoxifying, and cell metabolism-regulating properties [61]. In glutathione metabolism, we observed that DEGs were significantly downregulated in L016 vs. L016_BWS. In phenylalanine metabolism, DEGs were downregulated in L016_PDA vs. L016 probably due to the addition of valproic acid. Tryptophan is a precursor not only for protein synthesis but also for a variety of important biomolecules such as serotonin, and melatonin, tryptophan, and its metabolites play key roles in growth and development, gut–brain function, immune regulation, mitochondrial function, and energy metabolism [62].

Fungal polyketides include a large number of bioactive compounds ranging from drugs to harmful toxins [63]. Compared to *Aspergillus* and other ascomycetes, only very few polyketides have been isolated from basidiomycetes. However, as high-throughput sequencing of basidiomycetes has made genomic data more accessible, we are increasingly realizing that the ability of basidiomycetes to synthesize polyketides is more abundant than previously expected [63]. The main types of SM biosynthetic gene clusters in *P. portentosus* are terpenoids and Type I polyketides. The iterative Type I PKS ArmB of *A. mellea*, with the domains SAT-KS-AT-PT-ACP-ACP-TE, exhibits orsellinic acid synthase activity in vitro [63]. In this study, the PKS domains of *PpPKS1* and other NR-PKS in the same clade were similar to ArmB of *A. mellea*, but there were some differences in the modifier genes surrounding them, suggesting differences in the orsellinic acid biosynthesis gene clusters in different species. *PpPKS2* has a high homology with 6-Methylsalicylic acid from *S. pactum*, and it is hypothesized that *PpPKS2* synthesizes 6-Methylsalicylic acid. In *S. pactum*, a *β*-ketoacyl-ACP synthase (KAS) III-like protein (PtmR) has been identified, which functions as an acyltransferase that catalyzes the transfer of 6MSA to the aminocyclopentitol core. This enzyme is highly promiscuous, capable of utilizing several De-6MSA-pactamycin analogs as substrates to produce new derivatives of pactamycin [64]. De-6MSA-pactamycin was identified in the *P. portentosus* metabolome, and PtmR has been identified in the *P. portentosus* genome, which is presumed to be able to utilize De-6MSA-pactamycin to produce new pactamycin derivatives. Two new cytochalasans, flavichalasine N, flavichalasine O, together with six known cytochalasans, were isolated from *A. flavipes* [65]. In this study, the hybrid PKS-NRPS *PpPKS5* clustered with *A. flavipes* PKS, and Cytochalasins such as Cytochalasin E, Cytochalasin Z5, and xylarisin were detected in the *P. portentosus* metabolome, suggesting that *P. portentosus* PKS may synthesize cytochalasans. Interestingly, these compounds have not been previously reported to be isolated from *P. portentosus*, suggesting that their synthesis may be highly regulated. Although there is no complete cytochalasan gene cluster in the *P. portentosus* genome, possibly because the PKS genes in basidiomycetes do not exist in clusters, it still retains some important genes related to the production of cytochalasans, such as PKS-NRPS, trans-ER, and α/*β*-hydrolase. Genomic analysis can provide an in-depth understanding of the complete chemical characteristics of an organism, and some of the biosynthetic pathways encoded in the genome may help elucidate the bioactivity of SMs in *P. portentosus*.

Different cultivation methods can affect the production of fungal SMs as well as the expression of gene clusters, and the same gene may be expressed differently under different cultivation conditions, leading to the production of different compounds. Zhang et al. demonstrated that different cultivation methods affect the expression of PKS and TPS genes in *Taiwanofungus gaoligongensis*. The gene expression of *TgPKS1* and *TgPKS4* was significantly upregulated under solid cultivation conditions, while the expression of *TgPKS3*, *TgTRI5-1*, and *TgTRI5-2* was significantly upregulated under liquid cultivation conditions [66]. In this study, *PpPKS1* and *PpPKS2* gene expression was significantly upregulated under L016_BWS cultivation conditions, while the expression levels of *PpTRI5 1- 6*, *PpPtase*, *PpSTCs 1*, *5* and *7* and *PpSQS* genes were significantly upregulated in L016_BWS. In L016 liquid culture, *PpSTCs 2* and *4* and *PpPPS* gene expression was upregulated compared to L016_PDA and L016_BWS; *PpSQCY*, *PpPKS3*, *PpPKS4*, *PpSTCs 3*, *6* and *8* gene expression was upregulated compared to the liquid culture in solid culture condition (L016_PDA).

Co-regulation of TFs plays a central role in the secondary metabolic regulatory network of fungi [67]. For example, in *T. gaoligongensis*, the TFs *TgMYB9* and *TgFTD4* are involved in regulating the expression of the *TgPKS4* gene; *TgHOX1*, *TgHSF2*, *TgHSF3*, and *TgZnF4* synergistically act together to regulate the transcriptional activity of the *TgPKS3* gene, and *TgbZIP2* and *TgZnF15* regulate the expression of terpenoids [66]. The biosynthesis of aflatoxin and Sterigmatocystin is mainly regulated by the transcriptional activator AflR, a zinc-cluster TF with a specific DNA-binding domain that binds to specific sequences in the promoter region of genes to activate the expression of the aflatoxin cluster [68]. In this study, in combination with co-expression under different cultivation conditions and TF binding site prediction, it is hypothesized that the TFs *PpHSF1* and *PpHOX4* directly regulate the expression of *PpPKS1* by binding to the promoter region of the *PpPKS1* gene. The *PpPKS2* gene is regulated by the TF *PpTFIIB3*, and *PpPKS5* is regulated by the TF *Ppzf- C3HC4 28* regulation. In addition, *Ppzf-C2H2 32* positively regulates the expression of *PpSTCs 8*, *PpZn-clus 9* gene positively regulates the expression of *PpSTCs 6*, and *PpHSF5* positively regulates the expression of *PpSTCs 3*, thereby enhancing or activating the transcriptional process of TPS genes to promote the synthesis of TPS and, consequently, the synthesis of *β*-copaene.

## 5. Conclusions

This study integrated genomics, metabolomics, and transcriptomics to investigate the regulatory mechanisms of secondary metabolite biosynthesis in *P. portentosus* under different cultivation conditions. The genome of *P. portentosus* YAF023 (31.4 Mb, 15 scaffolds) was assembled using Illumina and Nanopore sequencing, revealing key functional genes, including 206 cytochrome P450s, 201 carbohydrate-active enzymes, 186 transcription factors, 18 terpene synthases (TPSs), and 5 polyketide synthases (PKSs). Multi-omics analysis identified specific biosynthetic pathways: *PpPKS1* likely contributes to Ethyl orsellinate production, *PpPKS2* and *PpPKS5* to 6-methylsalicylic acid and Cytochalasin Z5, respectively, *PpTRI5* to β-type trichodiene (a tetracyclic sesquiterpene), and PpSTCs to β-copaene analogs/derivatives. Regulatory network analysis predicted that *PpHOX4* and *PpHSF1* modulate *PpPKS1* expression, while *Ppzf-C2H2_32* and *PpHSF5* regulate *PpSTCs_8* and *PpSTCs_3*, respectively. This work establishes a key framework for advancing the exploration and utilization of bioactive secondary metabolites in *P. portentosus*.

## Figures and Tables

**Table 1:** *P. portentosus* genome assembly and functional annotation.

Item	Number	Item	Count	Percentage (%)
Total_length (bp)	31,421,828	All	7928	100.00
Scaffolds	15	Annotation	7749	97.74
GC_content (%)	48.91	KEGG	3112	39.25
N50 (bp)	2,638,669	Pathway	1702	21.47
N90 (bp)	1,761,355	Nr	7638	96.34
Average (bp)	2,094,788.53	Uniprot	7554	95.28
Median (bp)	2,550,121	GO	4981	62.83
Min (bp)	93,900	KOG	1625	20.50
Max (bp)	38,374,347	Pfam	5385	67.92
Total number of genes	7928	Interproscan	7305	92.14
The average cds_length of per gene (bp)	1500.19	Refseq	7398	93.31
The average exon_number of per gene	8.39	Tigerfam	1764	22.25

**Table 2:** The total number of CAZyme families in different fungal genomes.

Ecology Inches	Species	Genome Size (Mb)	No. of Genes	Accession Numbers	Total	AA	CBM	CE	GH	GT	PL
Ectomycorrhiza	*P. portentosus* YAF023	29.97	7928	JAYXKI000000000	201	42	4	8	89	53	5
	*P. portentosus* PP17026	32.74	9464	JAHRGP000000000	301	57	19	47	110	62	6
	*Paxillus ubicundulus*	53.01	22,354	JMDR00000000	273	47	12	39	108	61	6
	*Paxillus involutus*	58.30	17,984	JOMD00000000	404	62	14	64	171	84	9
	*Suillus luteus*	37.01	18,419	JMSM00000000	309	57	7	42	131	66	6
	*Pisolithus tinctorius*	71.01	22,845	JMDO00000000	241	42	2	38	87	69	3
Saprophytic	*Schizophyll umcommune*	38.48	13,189	ADMJ00000000	525	85	22	82	241	77	18
	*Lentinula edodes*	41.82	14,889	LDAT00000000	547	90	40	80	249	78	10
	*Volvariella volvacea*	36.45	11,084	AMXZ00000000	561	121	70	63	215	63	29
	*Coprinus cinereus*	38.70	16,862	JAAGWA000000000	567	132	67	86	192	74	16

Note. Data from Wan, J.-N (2021) [4].

**Table 3:** Classification of all metabolites identified in this study.

Classification of Metabolites	Positive Ion Mode	Negative Ion Mode
Organoheterocyclic compounds	164	103
Organic acids and derivatives	148	123
Lipids and lipid-like molecules	130	129
Benzenoids	85	71
Organic oxygen compounds	40	54
Phenylpropanoids and polyketides	36	48
Organic nitrogen compounds	31	2
Nucleosides, nucleotides, and analogs	24	14
Alkaloids and derivatives	16	1
Other	7	6
Unclassified	252	98
Total	933	649

**Table 4:** Binding sites of co-expressed PpTFs in the promoter regions of PpPKS and PpTPS.

TPS ID	Score	Relative Score	Sequence ID	Start	End	Strand	Predicted Sequence
PpHOX4	6.9166865	1	PpPKS1	573	576	+	TAAT
PpHSF1	11.563199	1	PpPKS1	2003	2009	+	ATGGAAC
Ppzf-C2H2 32	9.598243	1	PpSTCs 8	608	613	+	CCCCAC
PpHSF5	8.239277	1	PpSTCs 3	213	217	+	GGCC

## Data Availability

The genome sequence of *P. portentosus* YAF023 has been deposited at NCBI with accession number SAMN39487613, BioProject number PRJNA1066409, and SRA accession number SRR28483933 (GenBank accession number JAYXKI000000000) https://www.ncbi.nlm.nih.gov/datasets/genome/GCA_037892935.1/ accessed on 6 February 2025. The genome sequence of *P. portentosus* YAF023 has been deposited at NCBI with accession number SAMN39487613, BioProject number PRJNA1066409, and SRA accession number SRR28483933 (GenBank accession number JAYXKI000000000) https://www.ncbi.nlm.nih.gov/datasets/genome/GCA_037892935.1/ accessed on 6 February 2025.

## Supplementary Materials

The following supporting information can be downloaded at: https://www.mdpi.com/article/10.3390/jof11040323/s1. Table S1. qRT-PCR primers sequence; Figure S1. Species distribution of *P. portentosus* annotated by Nr database; Figure S2. Pfam domain distribution of genome annotation of *P. portentosus*; Figure S3. Clusters of orthologous groups of proteins (KOG) function classification of proteins in *P. portentosus*; Figure S4. Gene Ontology (GO) classification of *P. portentosus* genes; Figure S5. CAZyme functional classification chart of *P. portentosus*; Figure S6. PCA score plot of metabolite profiles of L016, L016_BWS, and L016_PDA of *P. portentosus*; Figure S7. Metabolome analyses of different cultivation conditions of *P. portentosus*; Figure S8. NR-PKS phylogenetic tree of *P. portentosus*; Figure S9. PR-PKS phylogenetic tree of *P. portentosus*; Figure S10. PKS-NRPS phylogenetic tree of *P. portentosus*; Figure S11. Structural characterization of the 13 TF Families in *P. portentosus*.
