# Supplementary material for: Genomic and Multi-Omics Analysis of Phlebopus portentosus: Effects of Cultivation on Secondary Metabolites
Source: J Fungi (Basel). 2025 Apr 18;11(4):323. doi: 10.3390/jof11040323 (PMC12028679; doi:10.3390/jof11040323)
Supplement: Supplementary file 1 [file jof-11-00323-s001.zip › jof-3491120-supplementary.pdf]

Table S1. qRT-PCR primers sequence.

Primer name	Sequences (5'~3')
Tubulin alpha F	TCGACAACGTCATGGACAAA
Tubulin alpha R	TGAGCAGTCGGAGTGCTCGA
PpPKS1F	ACATATCTTCGCATTCGAAC
PpPKS1R	GATGCTCGTGACTGACTTGA
PpHOX4F	TCAGCTTGCAGAGGATATCG
PpHOX4R	GATGACATGATATTCATGCG
PpPKS2F	CTGATACAGTACTTGGTCAT
PpPKS2R	CTGAACGATGTTGGGTTCGA
PpTFIIB3F	ATGAGCAGCTCCTCGTGAAG
PpTFIIB3R	TATAGGGTTGTTTGTCGTCT
PpPKS5F	CGACACGGCATGCTCTGCGT
PpPKS5R	TAGATAGAATCTCCGTCTTG
PpHSF5F	ATCTACGGCTTCATGCGCAA
PpHSF5R	TGTACATGTTTTGGTGGTGA
PpSTCs 6F	CTGACGCGGTCCAAGCTGGC
PpSTCs 6R	GGTATACGATCTTCCGAGCG
PpZn-clus 9F	ACAGAATAGCGGAGTTGGAA
PpZn-clus 9R	AGATATCCGTAATCGGTTCC
PpSTCs 8F	GTCATTCTGGACATGGAACG
PpSTCs 8R	ACGTATGCGACCACTTGACT

a

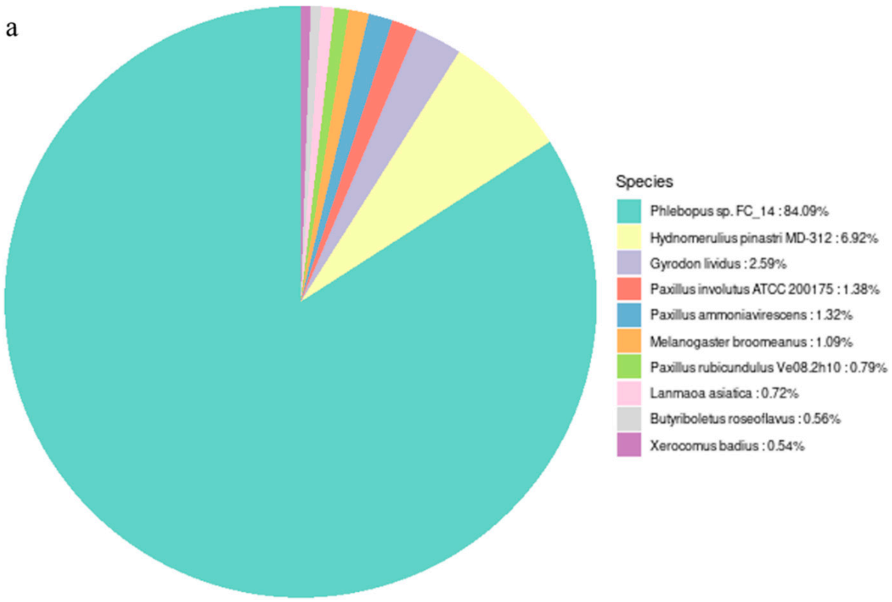

Figure S1. Species distribution of *P. portentosus* annotated by Nr database.

b

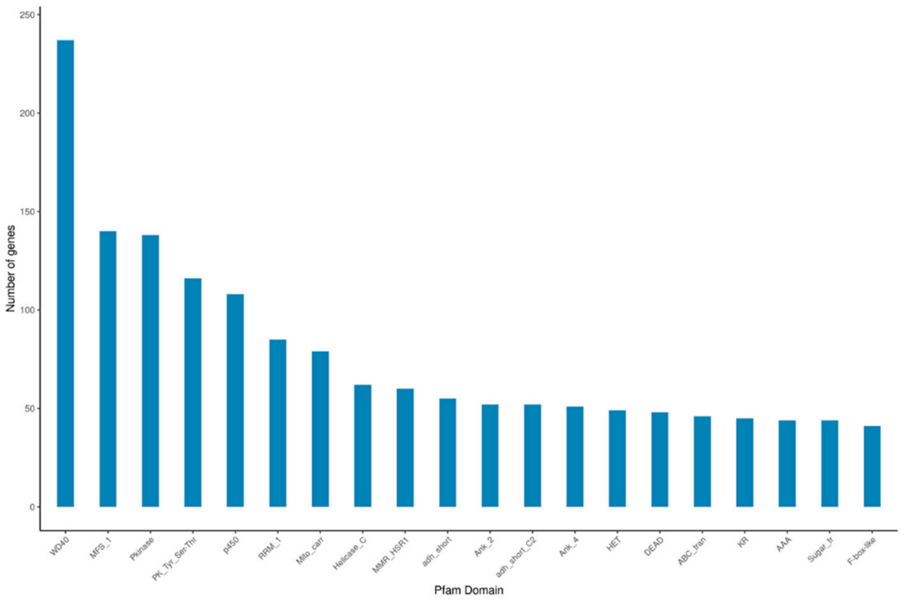

Figure S2. Pfam domain distribution of genome annotation of *P. portentosus*.

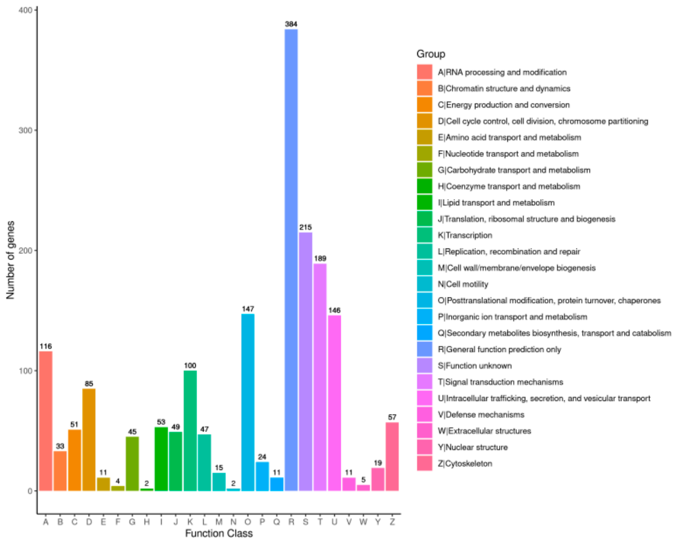

Figure S3. Clusters of orthologous groups of proteins (KOG) function classification of proteins in *P. portentosus*.

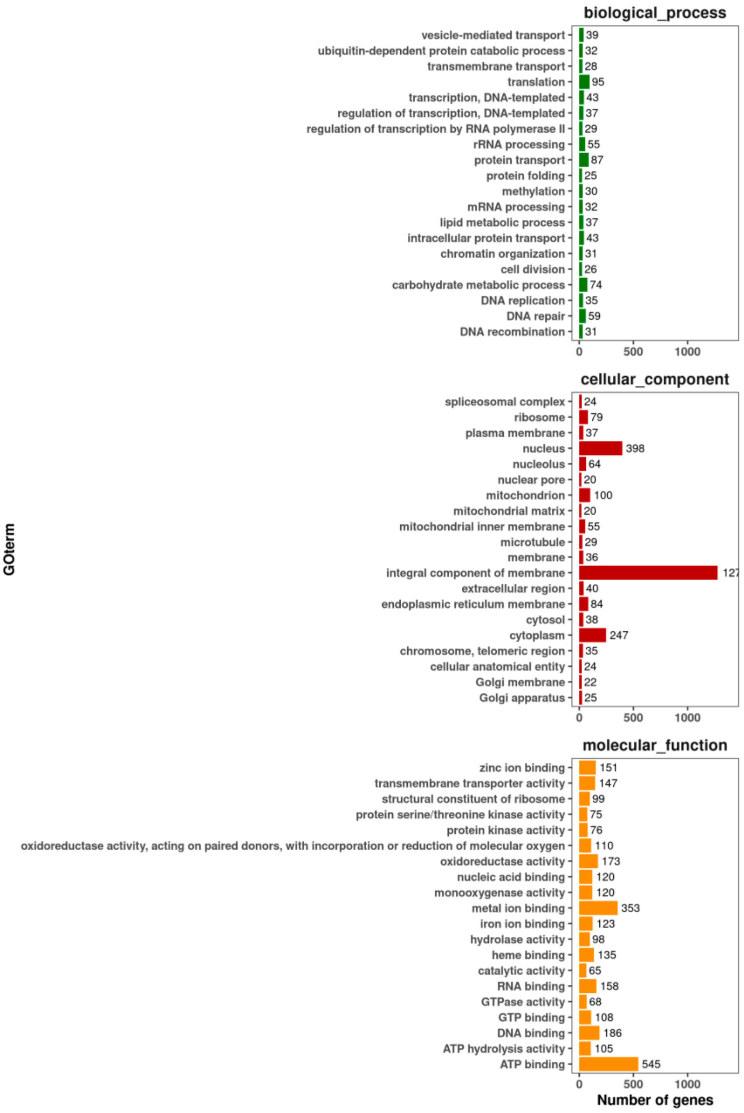

Figure S4. Gene ontology (GO) classification of *P. portentosus* genes. The major category biological process is colored by green, Cellular component by red and Molecular function by orange.

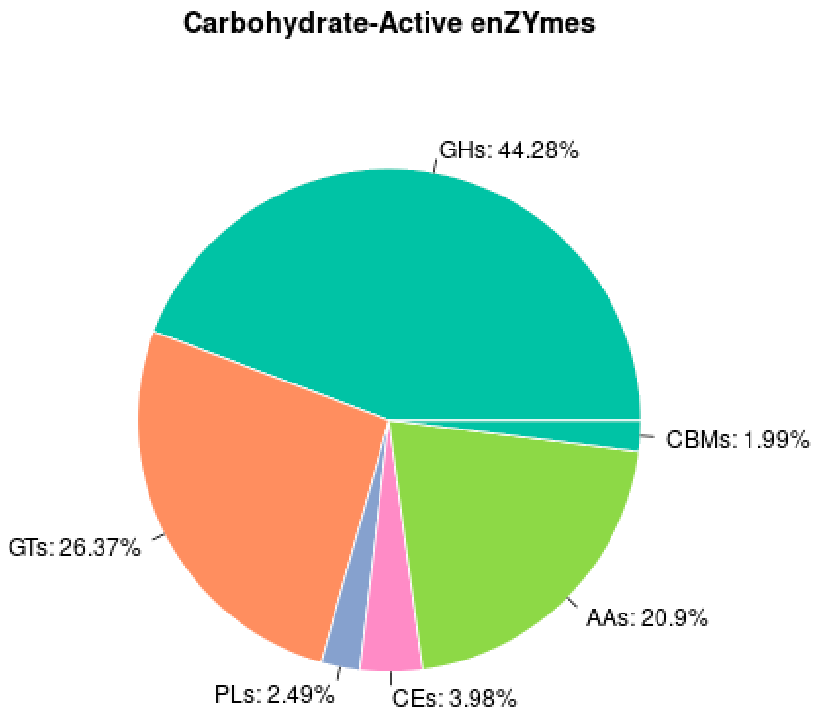

Figure S5. CAZyme functional classification chart of *P. portentosus*.

a

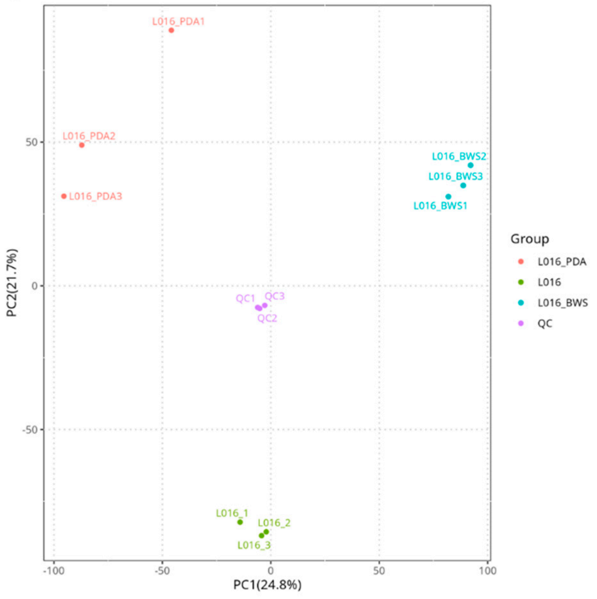

Figure S6. PCA score plot of metabolite profiles of L016, L016_BWS, and L016_PDA of *P. portentosus*.

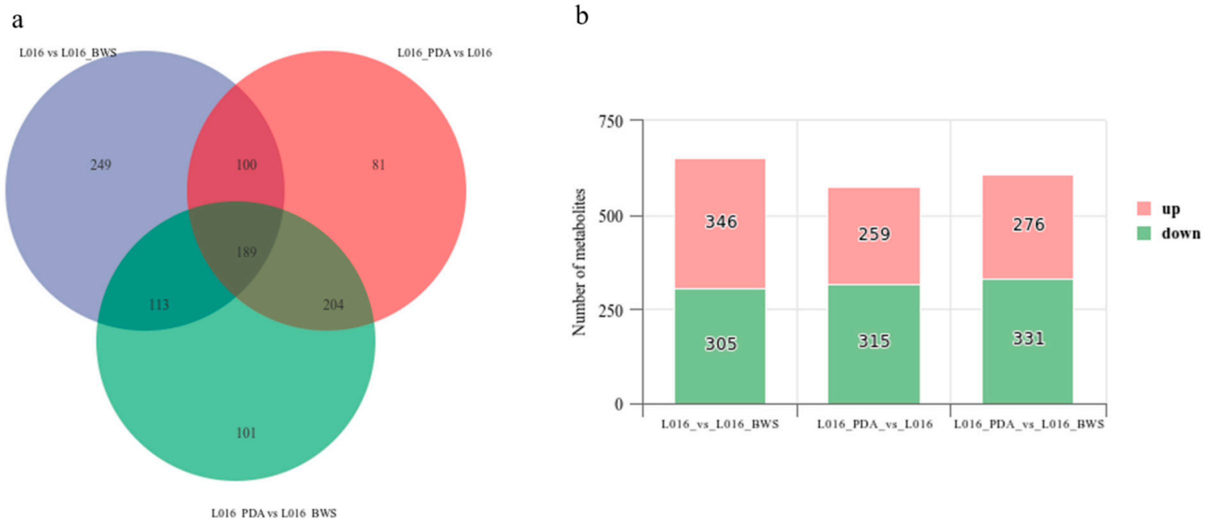

Figure S7. Metabolome analyses of different cultivation conditions of *P. portentosus*. (a) Venn diagram showing up- and downregulated DAMs based on pairwise comparisons of L016 vs L016_BWS, L016_PDA vs L016 and L016_PDA vs L016_BWS; (b) Bar graph of up- and downregulated DAMs based on pairwise comparisons.

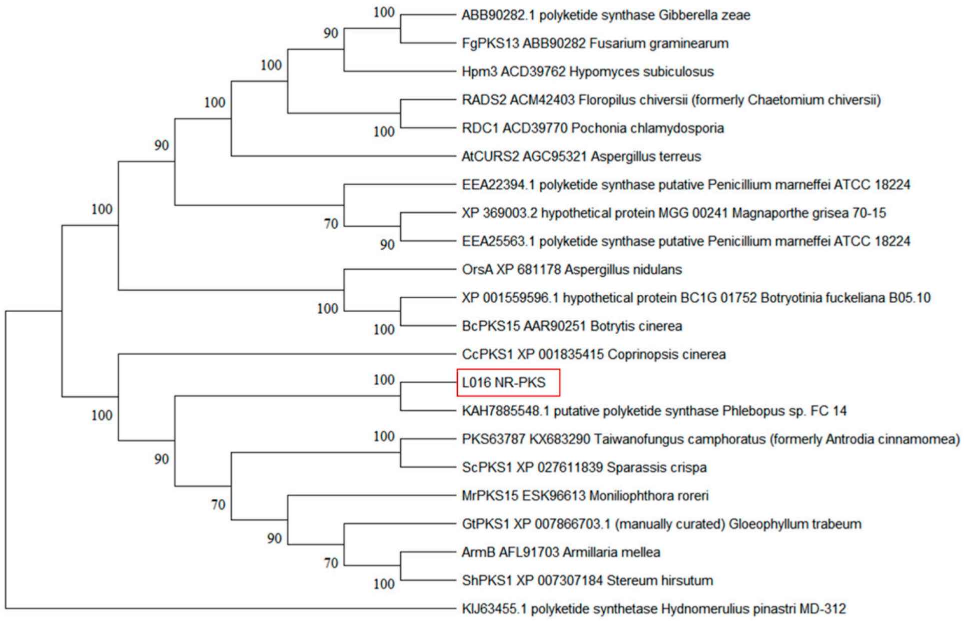

Figure S8. NR-PKS phylogenetic tree of *P. portentosus*. The ML tree was generated using MEGA11 with the JTT model. Values at the nodes represent the ML bootstrap proportions.

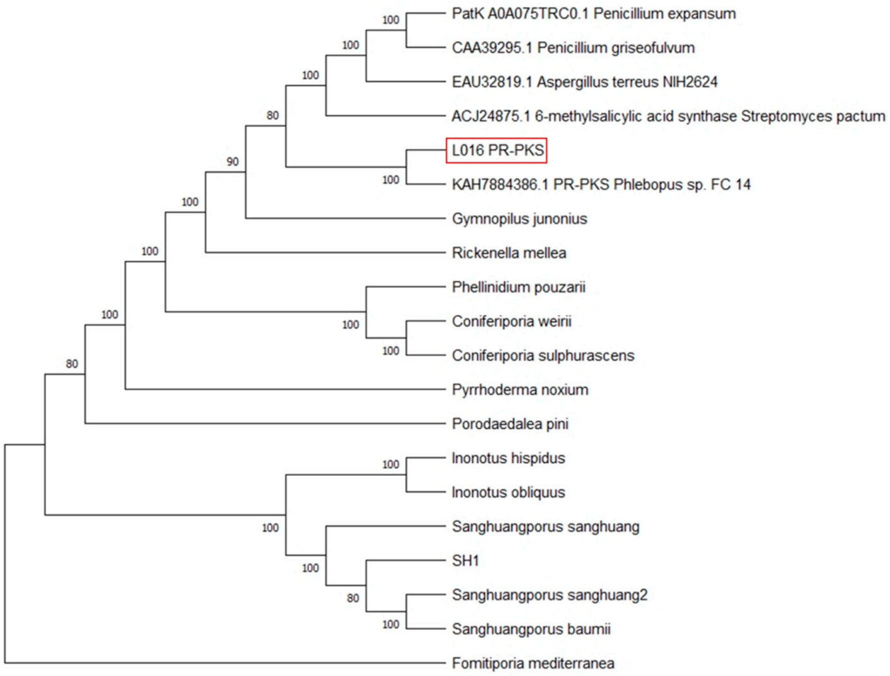

Figure S9. PR-PKS phylogenetic tree of *P. portentosus*. The ML tree was generated using MEGA11 with the JTT model. Values at the nodes represent the ML bootstrap proportions.

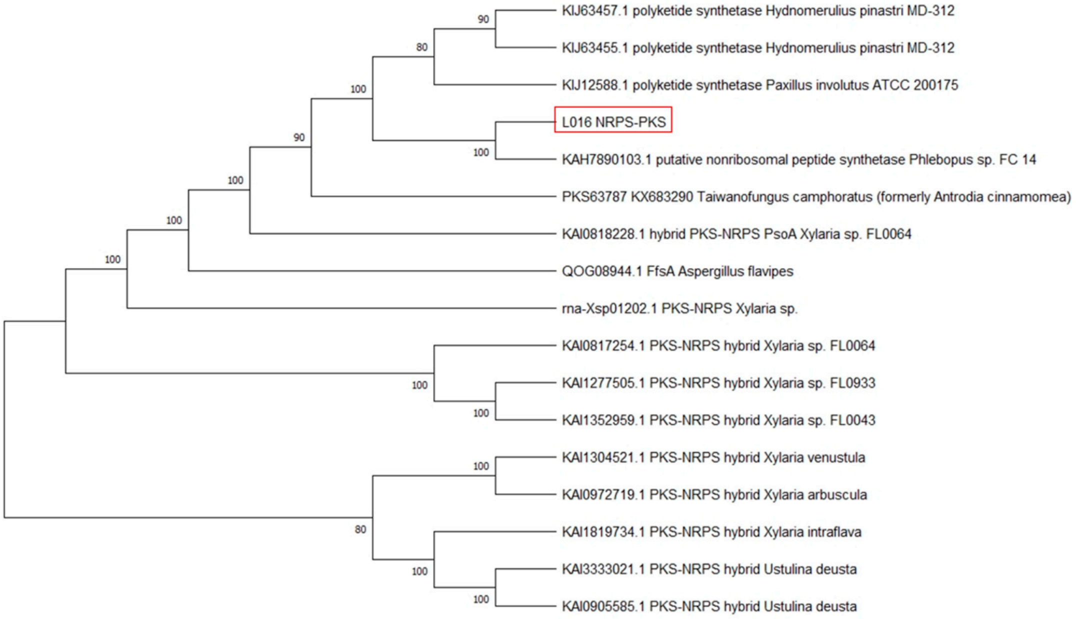

Figure S10. PKS-NRPS phylogenetic tree of *P. portentosus*. The ML tree was generated using MEGA11 with the JTT model. Values at the nodes represent the ML bootstrap proportions.

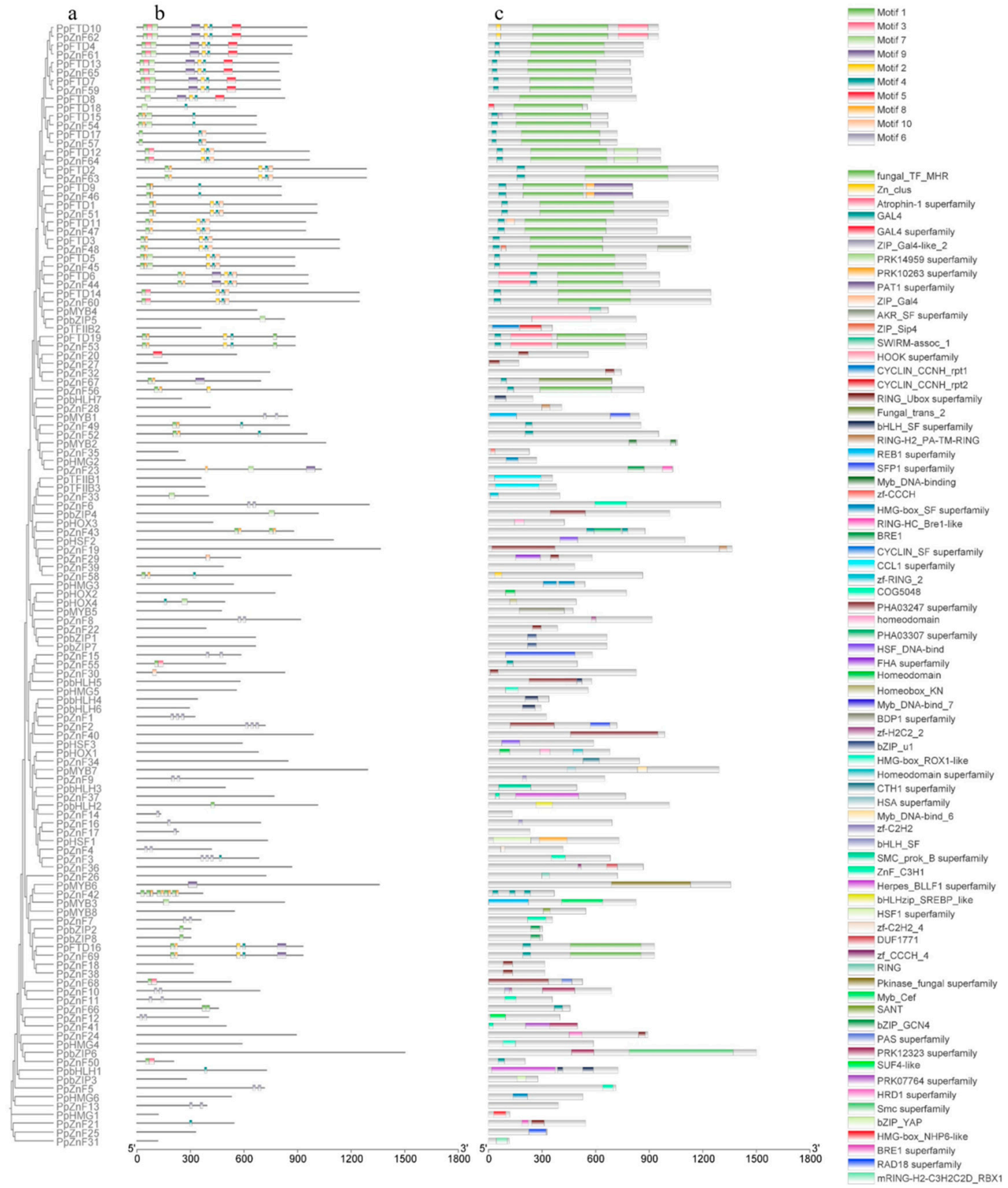

Figure S11. Structural Characterization of the 13 TF Families in *P. portentosus*. (a) Phylogenetic Tree of Protein; (b) Conserved Motif Analysi; (c) Conserved Domain Analysis.
